# Count data in biology—Data transformation or model reformation?

**DOI:** 10.1002/ece3.3807

**Published:** 2018-02-16

**Authors:** Anne P. St‐Pierre, Violaine Shikon, David C. Schneider

**Affiliations:** ^1^ Department of Ocean Sciences Ocean Sciences Centre Memorial University of Newfoundland St. John's NL Canada; ^2^ Department of Biology Memorial University of Newfoundland St. John's NL Canada

**Keywords:** coefficients estimates, count data, generalized linear model, model comparison, non‐normal error structure, Poisson distribution, residuals, transformation

## Abstract

Statistical analyses are an integral component of scientific research, and for decades, biologists have applied transformations to data to meet the normal error assumptions for *F* and *t* tests. Over the years, there has been a movement from data transformation toward model reformation—the use of non‐normal error structures within the framework of the generalized linear model (GLM). The principal advantage of model reformation is that parameters are estimated on the original, rather than the transformed scale. However, data transformation has been shown to give better control over type I error, for simulated data with known error structures. We conducted a literature review of statistical textbooks directed toward biologists and of journal articles published in the primary literature to determine temporal trends in both the text recommendations and the practice in the refereed literature over the past 35 years. In this review, a trend of increasing use of reformation in the primary literature was evident, moving from no use of reformation before 1996 to >50% of the articles reviewed applying GLM after 2006. However, no such trend was observed in the recommendations in statistical textbooks. We then undertook 12 analyses based on published datasets in which we compared the type I error estimates, residual plot diagnostics, and coefficients yielded by analyses using square root transformations, log transformations, and the GLM. All analyses yielded acceptable residual versus fit plots and had similar *p*‐values within each analysis, but as expected, the coefficient estimates differed substantially. Furthermore, no consensus could be found in the literature regarding a procedure to back‐transform the coefficient estimates obtained from linear models performed on transformed datasets. This lack of consistency among coefficient estimates constitutes a major argument for model reformation over data transformation in biology.

## 1 INTRODUCTION

As the analysis of variance (Fisher, 1925) came into wider use in the middle of the 20th century, attention turned to the assumptions (Eisenhart, 1947), the effects of violations of assumptions (Cochran, 1947), and remedies for violations (Bartlett, 1947) of the ANOVA assumptions of homogeneity, normality, and additive effects. In biology and ecology, experimental and observational studies often yield count data, which do not meet the assumptions for ANOVA (e.g., number of species in an area, number of offspring, number of colonies). In such instances, textbooks such as Sokal and Rolf 1969 and Zar 1974 recommend applying standard ANOVA procedures after addressing the assumption of homogeneity by application of a transformation appropriate to the assumed error distribution. With count data that follows a Poisson distribution, a square root transformation is recommended (Crawley, 2003; Maindonald & Braun, 2007; Sokal & Rohlf, 1969; Zar, 1974), while for datasets containing a large number of zeros, a square root transformation applied to *y* + 0.5 or to *y* + 3/8, where *y* is the response variable, may yield better results (Sokal & Rohlf, 1969). For data, where the variance is positively correlated with the mean, a logarithmic transformation is recommended (Sokal & Rohlf, 1969; Zar, 1974). When analyzing data bounded at zero and one, as with percentages and proportions or negative binomial counts, the arcsine square root transformation (arcsin(√*y*) where *y* is the response variable) is recommended (Anscombe, 1948; Sokal & Rohlf, 1969; Zar, 1974). Unfortunately, addressing homogeneity with an appropriate transformation does not necessarily address other assumptions (Bartlett, 1947; McCullagh & Nelder, 1983). For commonly used nonlinear equations in biology, the assumption of additive effects for count data can be addressed by linearization. The commonest examples (Crawley, 1993) are log transformation of the response variable for exponential relations (e.g., demographic rates), log transformation of the response and explanatory variables for power laws (e.g., species–area curves), and taking the inverse of the response and explanatory variable for simple asymptotic relations (e.g., the Holling “disk” equation), and log transformation for proportional change in discrete (count) data (Bishop, Fienberg, & Holland, 1974). The proliferation of special‐purpose transformations in the mid‐20th century culminated in the Box–Cox family of transformations, which puts a range of transformations on a single scale *Y* ^λ^, that includes inverse, log, square root, and power law transformations. This approach (Box & Cox, 1964) allows the contributions of additivity, homogeneity of variances, and normality to be separated.

As data transformation is an accessible solution to avoid non‐normal error distributions which allows analyses to be easily conducted by application of linear models while requiring limited computational power, it has been widely recommended in textbooks such as Sokal and Rohlf 1969 and Zar 1974, which have had a formative influence on the practice of statistics in biology and ecology. Data transformations can also be appealing as they can help decrease the impact of outliers and equalize the spread across different levels of a factor, thus improving linearity of the response variable and homogeneity of the variance. However, the approach is empirical; it does not address equations where the parameters are known from biological principles (Crawley, 1993, McCullagh & Nelder, 1983). Nor does the approach address the problem of biologically founded equations that are intrinsically nonlinear (Crawley, 1993), such as hyperbolic and asymptotic exponential equations. The use of transformations also produces parameter estimates which are hardly interpretable as they are no longer in the same scale as the original data. Log‐transforming data are known to produce erroneous (Currie & Schneider, 2011; Packard & Boardman, 2008; Stroup, 2013) estimates of linear trends and linear contrasts among means. With the addition of a fixed value for transformation of count data (e.g., using log(*y *+* *1) to work around the problem of log(0) for count data), these inaccuracies can be exacerbated (O'Hara & Kotze, 2010). Neither textbooks by Sokal and Rohlf 1969 nor Zar 1974 mention checking the residuals for normality before undertaking transformation, nor do these texts mention checking residuals after transformation to confirm that assumptions were met for calculating type I error rates to declare a statistical decision.

Landmark texts by Sokal and Rohlf 1969 and Zar 1974 follow Fisher 1925 in treating count data as a goodness of fit test where type I error in accepting one model over another is calculated from a χ^2^ distribution. McCullagh and Nelder 1983 introduced the generalized linear model (GLM), which extended Fisher's concept of likelihood to include transformation of both the response variable and the fitted value, the latter by specifying a link function. This approach is an alternative to transformations which allows the analyst to choose some combination of link function and error structure to address assumptions for estimating parameters and type I error where hypothesis testing is warranted. Thus, biological researchers can directly specify the error distribution and the relationship between the mean and the variance, thereby avoiding the inaccuracies that arise from transforming and back‐transforming data (O'Hara & Kotze, 2010). With the GLM approach, a binomial error structure is used for units scored in a binary fashion or as counts of successes relative to trials, and a Poisson or overdispersed Poisson error structure is used for counts per unit. This approach is now being extended (Stroup, 2013) to generalized linear mixed models (GLMM), which incorporate both random and fixed effects. GLMs function well on count data that include zeros (Bolker et al., 2008; McCullagh & Nelder, 1983); however, when there is an abundance of zeros relative to a Poisson or negative binomial error model, a hurdle or zero‐inflated model is recommended when fitting models and interpreting the estimates and trends (Lambert, 1992; Mullahy, 1986). The increasing availability of software and accessible texts (e.g., Dunteman & Ho, 2006; Hoffmann, 2004), combined with the advantages of avoiding transformation of data and back‐transformation of parameter estimates, has resulted in repeated recommendations to replace data transformation with GLM/GLMM procedures (Lo & Andrews, 2015; O'Hara & Kotze, 2010; Steel, Kennedy, Cunningham, & Stanovick, 2013; Warton & Hui, 2011; Wilson & Hardy, 2002).

Recently, Ives (2015) has argued for the traditional route of log‐transforming count data, as it has a lower rate of type I error (false positive) than other typical transformations (most textbooks suggest square root transformation for count data) or GLM. Ives (2015) showed that log‐transforming count data using the formula log(*y *+* *1) yields the best control over type I error, while acknowledging that potential inaccuracy in the estimates of coefficients was not considered. Ives’ (2015) conclusion is similar to earlier work by Stroup (2013), who showed that transformation and GLM/GLMM procedures are equivalent when the only concern is control of type I error, and thus, emphasis is only placed on the *p*‐value for declaring a statistical decision. Stroup (2013) also showed that transformations yield poor parameter estimates, especially in the case of the log transformation. Subsequently, Warton et al. (2016) used simulated overdispersed counts from an unbalanced sampling design to compare the outcomes of transformation and GLM with count data, arguing that GLM procedures should be applied rather than transformations if the GLM provides a good fit to the structure of the residuals, or if steps are taken to investigate and correct type I error (e.g., through resampling or permutation tests). Yet, Warton et al. (2016) underlined the importance of choosing an appropriate model based on data properties and diagnostic tools, which can be more difficult with small sample sizes. Warton et al. (2016) as well as Ives (2015) and Stroup (2013) used simulated data in their analyses, for which the error structure and true values of the parameter estimates are known; they did not extend their analyses to case studies where the error structure is unknown.

Given repeated recommendations to replace data transformation with GLM procedures, we investigated whether statistical textbooks for biologists continue to recommend data transformation for the analysis of count data. We also recorded the prevalence of data transformation versus model reformation in the peer‐reviewed literature over the period of 1980–2017, that is, when the majority of the textbooks consulted were published. We then extended the analysis of Stroup (2013), Ives (2015), and Warton et al. (2016) to the analysis of nonsimulated count datasets, using examples with a history of detailed treatment in textbooks. For 12 examples, we compared the residual plots, type I error rates, and coefficient estimates yielded by (1) a linear model after square root transformation of the response variable; (2) a linear model after log(*y *+* *1) transformation of the response variable; and (3) a GLM (with log link and either a Poisson or a negative binomial distribution). Given the prevalence of count data in ecological research, we focused the literature search and model comparisons to this type of data so that the results presented here are of interest to ecologists and biologist.

## 2 LITERATURE REVIEW

**Figure 1:**
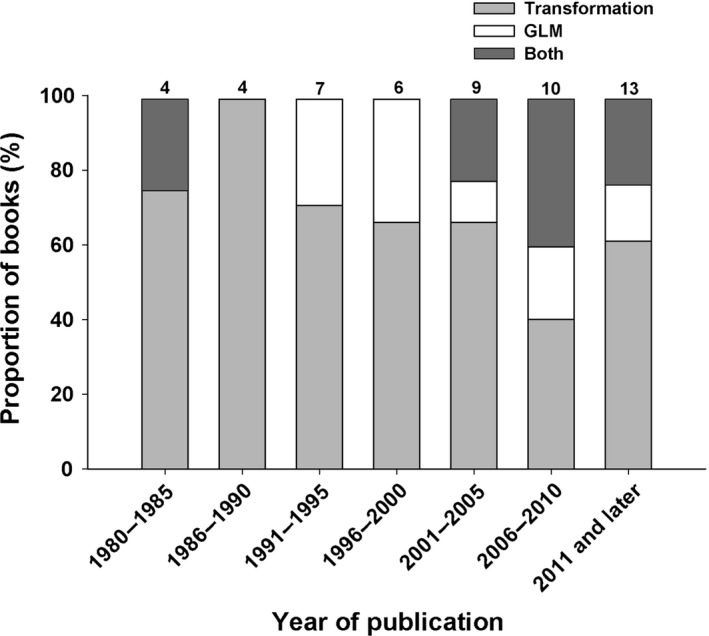
Proportion (%) of textbooks recommending the use of transformations (in gray), generalized linear models (GLM, in white), or both (in dark gray) when dealing with data with a non‐normal error structure. Data are presented by year of publication and grouped into 5‐year periods. All textbooks consulted were published between 1980 and 2017. The number above each bar represents the total number of textbooks in each time period

For the literature review, we divided our search into two categories: textbooks and journal articles published in the refereed literature. In the first, we consulted over 50 statistical textbooks published since 1980 (in addition to early editions of seminal textbooks by Fisher and Snedecor—see below) and noted whether the author(s) recommend the use of transformations or of GLM/GLMM when confronted with non‐Gaussian data (generally count data, proportions, or binary data). We focused our review on general‐purpose textbooks that are suitable for use in undergraduate and graduate levels statistics courses, as they are comprehensive in nature and represent that to which most students and researchers will be exposed. Hence, speciality textbooks focusing on GLM have been excluded, despite their abundance in recent years. The list of publications consulted and their recommendation for the analysis of data with non‐normal error structure is presented in Appendix S1. The results are summarized in Figure 1, where observations are grouped by 5‐year periods.

Data transformation to address problems of non‐normal or heterogeneous errors does not appear in any of the 12 editions of Fisher’ text (1925 through 1954). Nor does it appear in the first four editions of Snedecor (1937 through 1946).

Abnormality, non‐additivity, and heterogeneity of variance ordinarily appear together. It would be ideal if transformation could remedy all the difficulties, but that doesn't often happen. Snedecor 1937

The first recommendation for transformation that we could find in a text appears in Snedecor and Cochran (1967) with a brief treatment of the arcsine square root transformation for proportions (i.e., arcsin(√*y*) where *y* is the response variable). Text recommendations for the use of GLM begin with McCullagh and Nelder 1983 and continue in specialty texts, similarly focused on the GLM. Recommendations for transformation continued to appear in general‐purpose texts for biologists throughout the 20th century into the present (Figure 1, Appendix S1). Several textbooks consulted contained recommendations for both GLM and transformations when assumptions of the linear model were not met (one text between 1980 and 1985 (Atkinson, 1985), and nine texts since 2001, see Appendix S1). In all but one of these cases (Vittinghoff et al. 2012), transformations were suggested as an alternative only in cases where the use of a GLM did not meet the necessary assumptions. Despite some recommendations for the use of GLM in textbooks published since 1991, in addition to continued recommendations in speciality texts focused on the GLMs, transformations remain the predominant recommendation within general‐purpose texts. These results, and the recent publication of several journal articles debating the use of transformations and GLM (Ives, 2015; Lo & Andrews, 2015; O'Hara & Kotze, 2010; Warton & Hui, 2011; Warton et al., 2016), show that there is not yet a consensus in general‐purpose statistical texts directed toward biologists and ecologists as to the best practice for dealing with non‐normal residuals.

**Figure 2:**
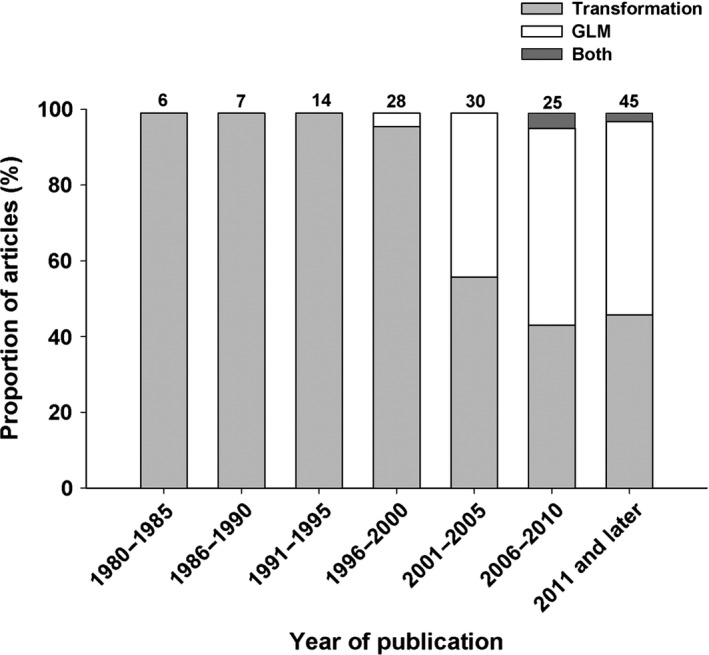
Proportion (%) of research articles using transformations (in gray), generalized linear models (GLM, in white), or both (in dark gray) when dealing with data with a non‐normal error structure. All articles considered come from peer‐reviewed journals in the fields of biology and ecology. Data are presented by year of publication from 1980 to 2017, grouped into 5‐year periods. The number above each bar represents the total number of articles in each time period

For the second part of our literature review, we used Google Scholar to find articles published in peer‐reviewed journals in the fields of biology and ecology presenting original research in which analyses are conducted on count data and determined whether the authors had transformed their data or applied a GLM/GLMM. We used the keywords “count” or “count data” and focused on articles published after 1980. In order to cover both terrestrial and marine publications, we concentrated our efforts on ten ecology journals that publish mainly original research and are influential in their respective fields (as marked by their relatively high impact factor score and citations): *Ecology*,* Oikos*,* Journal of Animal Ecology*,* Ecology Letters*,* Plant Biology*,* Journal of Ecology*,* Nature*,* Plant Ecology*,* Marine Biology*, and *Marine Ecology Progress Series*. The list of publications consulted and the statistical method applied in each (either transformations or GLM/GLMM) is presented in Appendix S2. The results are summarized in Figure 2, where observations are grouped by 5‐year periods.

The results of the literature review of journal articles show that all the articles published before the year 1996 used the classical approach of applying a transformation to the response variable prior to analysis. The proportion of articles using GLM increased in the following decades: from 4% of the publications using these models between 1996 and 2000, to 43% between 2000 and 2005, and to approximately 52% after 2006. This trend may be due at least in part to the increasing availability of software packages that allow easy application of GLM/GLMM and to increasing knowledge of these software among researchers. It may also be due to the rise in number of articles critiquing the use and outcomes of data transformation, as discussed in our introduction. However, the increasing number of researchers (as indicated by articles published in the primary literature) favoring GLM/GLMM over transformation cannot be linked to general‐purpose texts, which continue to recommend transformation (Figure 1, Appendix S1). It is possible that researchers rely more heavily on the documentation related to their preferred software or on the increasing number of textbooks specialized in the application of GLM/GLMM to guide their analysis, rather than on general‐purpose textbooks. Researchers, in particular graduate students, also rely heavily on web resources such as online course material and tutorials to guide the execution of analyses on their favorite software. Of all the journal articles consulted, only two mentioned using both GLMs and transformations (Langwig et al., 2012; McCauley et al., 2010); for certain data in their analyses, the GLM did not meet the necessary assumptions and thus a transformation was applied, as was suggested in nine textbooks consulted in our review (see Appendix S1).

## 3 MODEL COMPARISONS

### 3.1 Method

To contrast the difference in results when using transformation (square root and log) and reformation (GLM), we applied and compared different models to 12 analyses of count data obtained from the landmark text of McCullagh and Nelder 1983 and from the widely cited text of Agresti (1996). These datasets were chosen as their error structure fitted well a Poisson distribution, hence eliminating the risk of differences caused by a poor choice of error structure. In addition, the datasets are not overdispersed (except in one case, see Appendix S6). In each case, the assumption of homogeneity of variances was assessed graphically (Neter & Wasserman, 1974). All analyses were run using R 3.2.2 with the car and MASS packages (R Development Core Team, 2009).

Each dataset was analyzed in three different ways by (1) applying a square root transformation to the response variable and running a linear model; (2) applying a log transformation (log(*y *+* *1) where *y* is the response variable) and running a linear model, and (3) running a GLM with either a Poisson or negative binomial distribution, depending on which was more appropriate from examination of residual plots. The residual versus fit plots, the outcome of the test statistic (significance of the *p*‐value), and the value of the coefficients (means for ANOVA factors, and slopes and intercepts for regressions) were compared among the models applied to each dataset. The models for each dataset, as well as the resulting comparisons, are presented in Appendices S3–S14.

To obtain estimates of the coefficients (means or intercept and slope, for categorical or regression analyses, respectively) from the linear model applied to the square‐root‐transformed data, a back‐transformation was necessary. For categorical analyses, we first obtained the estimate of the “transformed” mean for each factor by calculating the sum of each factor added to the value of the intercept estimate, as is the procedure for calculating means from the estimates of a model with the identity link (Gaussian/normal distribution). Then, the value of each “transformed” mean was squared to return the values to the original scale. In the case of regressions, the “transformed” estimates of the intercept and the slope were squared to place them back on the original scale, and when the uncorrected estimate was below zero, the negative sign was retained after squaring to maintain the same trend (e.g., increasing or decreasing slope, positive or negative intercept).

A back‐transformation was also applied to the estimates of the coefficients of the linear model applied to the log‐transformed data. For categorical data, we first obtained the estimate of the “transformed” mean for each factor by calculating the sum of each factor (separately) and the “transformed” estimate of the intercept. Then, each “transformed” coefficient (β) was back‐transformed by exponentiating it and subtracting 1 (i.e., e^β^ − 1) to obtain the geometric means. The same method (i.e., exponentiating the estimate and subtracting one) was used to obtain the value of the intercept and the slope in the case of regressions.

The values of the coefficients for the GLM were easily obtained and are known to be accurate (Stroup, 2013; table 11.1). First, we exponentiated the estimates to obtain the coefficients for each factor, then we multiplied the coefficient of each factor by the coefficient of the intercept (the mean) to obtain the mean for each factor (i.e., e^β^ · β_intercept_). We followed this procedure because the log link was used in the GLM, which produces coefficients that are multiplicative in nature, as the coefficients provide a proportion relative to the intercept mean. In the case of regressions, the exponentiated coefficient for the regression factor shows the proportion change with each increment of the explanatory variable. Thus, we obtained the value of the slope, which was necessary for comparison with the other two models, by subtracting one from the value of the proportion change (i.e., the value of the exponentiated coefficient).

### 3.2 Comparison of the residual plots

**Figure 3:**
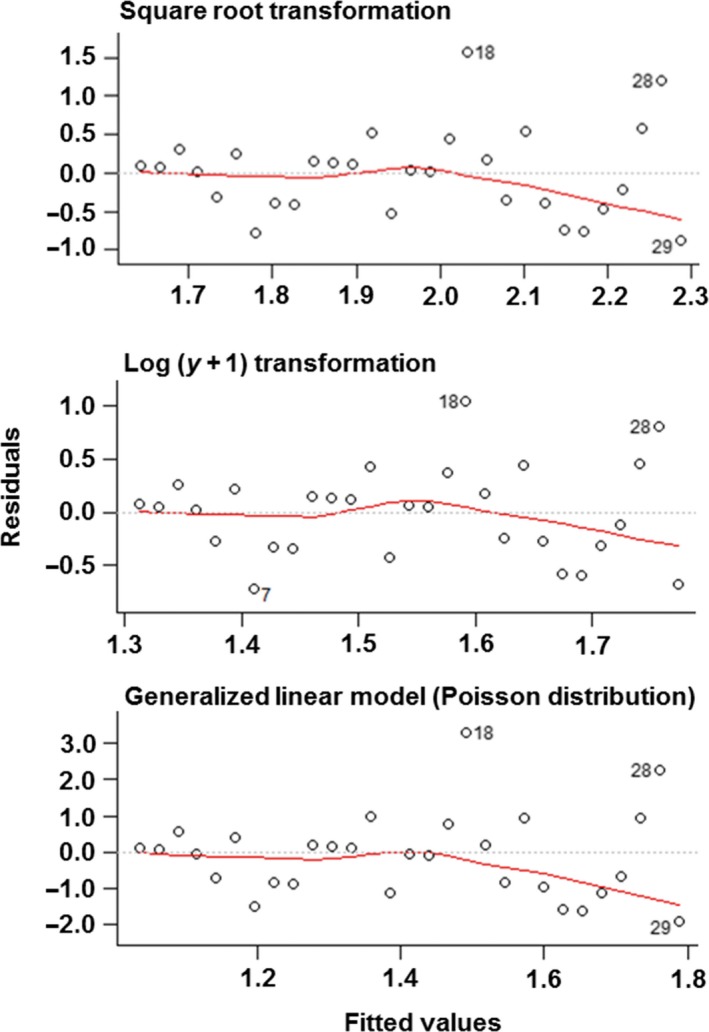
Example of comparison of residual versus fit plots for (top to bottom) linear model performed on square‐root‐transformed data, linear model performed on log‐transformed data (using log(*y* + 1) where *y* is the value of the response variable), and generalized linear model, applied to the dataset presented in Appendix S6, representing the number of train‐to‐car collisions in relation to the year (from Agresti, 1996, p. 83). Red lines represent smoothed curves fitted to the data in each plot. See Appendices S3–S14 for residual versus fit plots for all 12 datasets used in case studies

For eight of the 12 datasets, the residual versus fit plots were very similar between the three models—two transformations and one GLM (Figure 3). When a difference was observed, it was almost always in the linear model applied to square‐root‐transformed data, which displayed more of a fan‐shaped trend than the plots from the other two models (in three datasets of 12, see Appendices S9–S11). However, in most cases, all three models had acceptable residual versus fit plots (i.e., uniform bands in the plots).

### 3.3 Comparison of the outcome of the statistical test

The outcome of the statistical test was generally consistent among the three models for a given dataset. Although the actual *p*‐value varied substantially between the models, only in the case of two datasets (out of 12, see Appendices S6 and S13) did this variation result in a change of decision at a fixed tolerance for type I error of α = 0.05, as both models using transformed data yielded a different outcome compared to the GLM.

### 3.4 Comparison of the coefficient values

**Figure 4:**
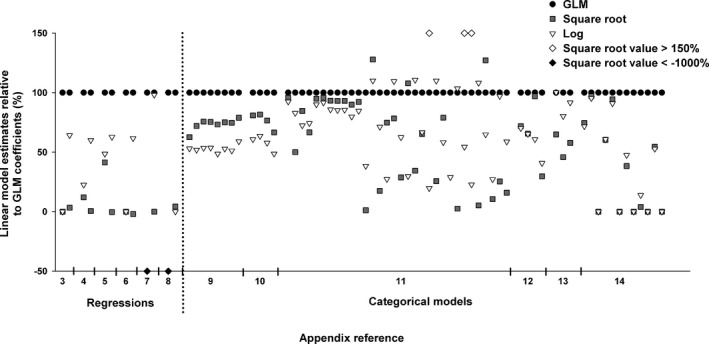
Linear model estimates relative to those calculated in the generalized linear models (GLM) (% difference) for each dataset. Complete results are presented in Appendices S3–S14

Given that the estimates obtained by application of the GLM on untransformed data are known to be accurate (Stroup, 2013; table 11.1), the estimates obtained by the models applied to transformed data were compared to those of the GLM to evaluate their accuracy. Both models using the transformed data underestimated the value of the intercept and slope in regressions compared to GLM estimates (Figure 4, Appendices S3–S8). In these models, the square root transformation typically had the lowest values of the three models, yielding an estimate more distant from the accurate values obtained with the GLM on untransformed data than the estimate calculated for the log transformation. Similarly, in models containing categorical explanatory variables, we found that the coefficients calculated for linear models applied to transformed data were generally lower than the ones obtained from the GLM (Figure 4, Appendices S9–S14). In contrast to the regression models, the coefficients of the square‐root‐transformed data were not consistently lower than those of the models using the log transformation.

## 4 DISCUSSION

The proportion of journal articles in the primary literature in which authors used reformation (GLM/GLMM) has steadily increased over the past 25 years (Figure 2) relative to the articles in which authors transformed their data to then apply a linear model. However, this trend in the primary literature does not represent the recommendations found in general‐purpose texts for biologists (see Figure 1). The increasing use of GLM/GLMM procedures to address non‐normal errors may be consistent with the increase in special‐purpose texts, but was not consistent with the continuing recommendation of transformation in textbooks for biologists in general and ecologists in particular.

Ives (2015) argued that the traditional approach of transformation is preferable as GLMs are prone to high type I error rates under some simulated conditions. Stroup (2013, table 11.1) similarly reported a slightly higher rejection rate for a GLM with Poisson error than for log‐transformed data. Stroup (2015) attributed the disparity to excessively conservative tests with log transformation. In addition, Stroup (2013) found that the log transformation did a poor job estimating parameters. Also, the log transformation performed poorly as measured by disparity between “the standard error of the estimator and the standard deviation of the estimator's sampling distribution” (Stroup, 2013; p. 340). It has been demonstrated repeatedly that the GLM produces narrower confidence limits than transformation of the response variable (Hamada & Nelder, 1997; Lewis, Montgomery, & Myers, 2001). When we extended the analysis of data transformation versus reformation from simulated data with known parameters to datasets with unknown parameters, we found that the residuals differed little between linear models applied to transformed data and GLM. Consistent with the findings of Stroup (2013) for simulated data, we found that decisions from the *p*‐values (at α = 0.05) were usually consistent among models (square‐root‐transformed data, log‐transformed data, and GLM), although GLMs can be prone to high type I error rates if misspecified (Ives, 2015).

As expected from previous studies, the coefficients from the linear models run on transformed data, once back‐transformed, were consistently distant from the estimates on the original scale obtained with the GLM (Figure 4). The distance of the coefficients of the transformed datasets relative to the accurate coefficients from the GLM (Stroup, 2013) varied dramatically from one dataset to another. This suggests that a general procedure to correct for such inaccuracy cannot be applied. A primary source of uncertainty in our procedure lies in the method used for back‐transforming the coefficients obtained from the square‐root‐ and log‐transformed data. Despite extensive search in statistical textbooks (Appendix S1) and online statistical resources, there was insufficient and often conflicting information on the appropriate procedure to apply. Little information on the procedure to back‐transform estimates was found in statistical textbooks aimed toward biologists and ecologists, and the few recommendations presented typically addressed only simple scenarios. For example, one squares the estimates to obtain the coefficients with square‐root‐transformed data. However, because this procedure will always produce positive coefficients, the negative sign must be reapplied as appropriate, yet this correction was never stated explicitly. With regard to log transformation of count data, back‐transformation was calculated in the literature as 10^β^. However, due to the occurrence of zeroes in count data, a constant was generally added to the data prior to applying the log transformation (e.g., log(*y *+* *1)). Nowhere in the literature did we find a clear preference for using 10^β^ − 1 as opposed to 10^β − 1^ to back‐transform. The lack of a clearly stated procedure for back‐transformation of estimates adds an additional level of uncertainty when comparing results from different studies. For the analyses presented here, coefficients were back‐transformed that seemed most logical based on the overall recommendations found through a thorough web search and consultation of statistical textbooks. We do not maintain that our approach to back‐transformation is definitive. Other investigators attempting back‐transformation of coefficient estimates might well arrive at a different choice in methods and thus obtain different outcomes, which points to a clear need for a definitive treatment of the problem of accurate back‐transformation of coefficient estimates. The analysis of data on its original scale, either via randomization tests or by use of an appropriate error model, is an established and certain way of yielding unambiguous parameter estimates in the field of quantitative biology. The uninterpretability of transformed data has been repeatedly stated (Warton & Hui, 2011). The hidden cost of transformation is that parameter estimates are no longer representative measurements, defined by Krantz, Luce, Suppes, and Tversky (1971) as mappings from empirical relations into numerical relations. A transformed variable ceases to represent additive empirical relations, such as 2 m + 2 m = 4 m. Put another way, units are lost in transformation. Cases where representational measurement is preferable are readily imagined.

Data transformation can be applied for four purposes, as stated by Crawley (1993, p. 214): (1) to obtain a constant error variance; (2) to obtain approximately normal errors; (3) to achieve linear (additive) relation between response and explanatory variables (but note that while some transformations lead to additive relations [such as log(proportions)], transformations destroy additivity on the original scale of measurement); and (4) to allow more straightforward scientific explanation. The first two establish sound estimates of type I error. The second and third establish the applicability of model results to the analytic goals. However, achieving all four purposes is rare; trade‐offs are almost inevitable. Given our results, where the use of GLM had minimal impacts on the residuals and the statistical outcome, and yielded interpretable coefficients, we take into account the advantages and trade‐offs of both analytical methods (transformation and GLM) and recommend that transformation be used only in cases where (a) type I error is more important than parameter estimates, (b) an estimate of type I error is necessary, or (c) a suitable error model cannot be identified from examination of residual plots and other diagnostics.

Our recommendation (a) addresses the trade‐off between control over type I error and interpretability of coefficient estimates. When might type I error be more important? A good example comes for Fisher's 1925 text: prevalence of typhoid in uninoculated and inoculated soldiers. The parameter estimate (an odds ratio) is less important than decision based on type I error, in the context of the cost of inoculation versus the cost of mortality under trench warfare conditions in World War I. Our recommendation (b) pertains to cases when type I error estimates is necessary for planning experiments, designing monitoring programs, and justifying research proposals. In many fields, including some areas of ecology, calculations of adequate sample sizes, relative to standards for type I and type II errors (typically 5% and 20%, respectively), are expected. Lastly, physically or biologically interpretable regression estimates used for interpolation are of little value if the error model employed to produce the estimates does not fit the data (i.e., a suitable error model cannot be identified from examination of residual plots and other diagnostics, our recommendation (c)). In such cases, it would be better to get the type I error correct via heuristic transformation of the response variable rather than focusing on coefficient estimates.

## 5 CONCLUSIONS

Although the transformation of data and the use of GLM/GLMM both continue to be recommended in statistical textbooks from 1967 to the present, the occurrence of GLM/GLMM applied to count data in peer‐reviewed articles has shown a clear increase since 2000. Our findings from 12 analyses with unknown parameters extend those of Stroup (2013): Transformation and GLM techniques applied to count data yielded *p*‐values that rarely resulted in a change in the statistical decision, and the use of transformations resulted in inconsistent and sometimes large differences in the coefficient estimates. The absence of a consensus or clear procedure regarding the back‐transformation of coefficient estimates is at once a gap in statistical practice and a major argument against the use of transformations. GLM/GLMM methods yield directly interpretable estimates on an additive rather than multiplicative scale, without greatly impacting the control over type I error in our analyses as well as in synthetic data sets with known parameters (Stroup, 2013). Furthermore, these estimates are free of the known inaccuracy present in parameters estimated on a transformed scale, as occurs with data transformations. Measures can also be taken to improve control over type I error in GLM procedures, for example, through permutation or parametric bootstrap (Warton et al., 2016). Given the availability of statistical software capable of running such models, together with accessible texts, we recommend the use of model reformation over data transformation for the analysis of count data, except when a decision against a fixed type I error rate is more important than estimating a parameter on its original scale.

## CONFLICT OF INTEREST

None declared.

## DATA ACCESSIBILITY

All data used in this study was obtained from published sources, that is, statistical textbooks by McCullagh and Nelder 1983 and Agresti (1996). Further information regarding the specific datasets studied is available in Appendices S3–S14. A template of the R script used in the data analysis is also presented in Appendix S15.

## AUTHORS’ CONTRIBUTION

A. P. St‐Pierre and V. Shikon equally contributed to the literature reviews and statistical analyses, under guidance and supervision of D. C. Schneider. A. P. St‐Pierre oversaw the manuscript preparation and editing. All authors contributed to project design and manuscript preparation.
