# Supplementary material for: Count data in biology—Data transformation or model reformation?
Source: Ecol Evol. 2018 Feb 16;8(6):3077–85. doi: 10.1002/ece3.3807 (PMC5869353; doi:10.1002/ece3.3807)
Supplement: Supplementary file 1 [file ECE3-8-3077-s001.docx]

**Appendix 1**

**Review of recommended procedures to apply for data with a non-normal error structure, as presented in textbooks published between 1980 and 2017**

**Table 1**. Overview of books published between 1980 and 2017, with their recommendation of the use of transformations (T) or generalised linear model (GLM) approaches when analysing data with a non-normal error structure given the parameters.

| **Authors** | **Title** | **Publisher** | **Year** | **Procedure** |
| --- | --- | --- | --- | --- |
| Bishop | Statistics for biology (3^rd^ edition) | Longman | 1980 | T |
| Finney | Statistics for biologists | Chapman & Hall | 1980 | T |
| Ripley | Spatial Statistics | Wiley | 1981 | T |
| Atkinson | Plots, Transformations, and Regression - An introduction to graphical methods of diagnostic regression analysis | Clarendon Press | 1985 | T and GLM |
| Damon & Harvey | Experimental design, ANOVA, and regression | Harper & Row | 1987 | T |
| Snedecor & Cochran | Statistical Methods (8^th^ edition) | Iowa State | 1989 | T |
| Campbell | Statistics for biologists (3^rd^ edition) | Cambridge University Press | 1989 | T |
| Bowerman & O'Connell | Linear statistical models - An applied approach (2^nd^ edition) | PWS-KENT Publishing Company | 1990 | T |
| Brown & Rothery | Models in biology: mathematics, statistics, and computing | John Wiley and sons | 1993 | GLM |
| Norman & Streiner | Biostatistics - The bare essentials (2^nd^ edition) | BC Decker | 1993 | T |
| Watt | Introductory statistics for biology students | Chapman & Hall | 1993 | T |
| Fry | Biological data analysis - A practical approach | Oxford University Press | 1993 | T |
| Clarke | Statistics and experimental design | Edward Arnold | 1994 | T |
| Daniel | Biostatistics: a foundation for analysis in the health sciences (6^th^ edition) | Wiley | 1995 | Logistic Regression |
| Bailey | Statistical Methods in biology (3^rd^ edition) | Cambridge University Press | 1995 | T |
| Zar | Biostatistical analysis (3^rd^ edition) | Prentice Hall | 1996 | T |
| Agresti | An introduction to categorical data analysis | Wiley | 1996 | GLM |
| Gardiner | Statistics for the biosciences | Prentice Hall | 1997 | T |
| Rao | Statistical research methods in the life sciences | Duxbury | 1998 | T |
| Sprent | Data driven statistical methods | Chapman and Hall | 1998 | GLM |
| Norman & Streiner | Biostatistics - The bare essentials (2^nd^ edition) | BC Decker | 2000 | T |
| Scheiner & Gurevitch | Design and analysis of ecological experiments | Oxford | 2001 | T |
| Grafen & Hails | Modern Statistics for the Life Sciences | Oxford | 2002 | T |
| Quinn & Keough | Experimental design and data analysis for biologists | Cambridge University Press | 2002 | T and GLM |
| Blaesild & Granfeldt | Statistics with applications in biology and geology | Chapman & Hall/CRC | 2003 | T and GLM |
| van Emden | Statistics for terrified biologists | Blackwell Publishing | 2003 | T |
| Hocking | Methods and applications of linear models - Regression and the analysis of variance (2^nd^ edition) | Wiley | 2003 | T |
| Selvin | Biostatistics: how it works | Pearson Prentice Hall | 2004 | GLM |
| Gotelli & Ellison | A primer of ecological statistics | Sinauer Associates, Inc | 2004 | T |
| Kutner, Nachtsheim, Neter & Li | Applied Linear statistical models (5^th^ edition) | McGraw-Hill Irwin | 2005 | T |
| Clark | Models for ecological data - An introduction | Princeton University Press | 2007 | GLM |
| Gelman & Hill | Data analysis using regression and multilevel/Hierarchical models | Cambridge University Press | 2007 | T and GLM |
| Zuur, Ieno & Smith | Analysing Ecological Data | Springer | 2007 | T and GLM |
| Norman & Streiner | Biostatistics - The bare essentials (3^rd^ edition) | BC Decker | 2008 | T |
| Gillman | An introduction to mathematical models in ecology and evolution - Time and space (2^nd^ edition) | Wiley Blackwell | 2009 | T |
| Hilbe | Logistic regression models | CRC Press | 2009 | GLM |
| Le | Health and numbers: A problems-based introduction to biostatistics (3^rd^ edition) | Wiley-Blackwell | 2009 | T |
| Zuur, Ieno & Meesters | A beginner's guide to R | Springer | 2009 | T |
| Heiberger & Holland | Statistical analysis and data display: an intermediate course with examples in S-plus, R, and SAS | Springer | 2010 | T and GLM |
| Logan | Biostatistical design and analysys using R | Wiley Blackwell | 2010 | T and GLM |
| Morris | Design of Experiments: An introduction based on linear models | CRC press | 2011 | T |
| Rosner | Fundamentals of biostatistics (7^th^ edition) | Brooks/Cole Cengage Learning | 2011 | T |
| Sokal & Rohlf | Biometry (4^th^ edition) | Freeman | 2012 | T, and Logistic regression |
| Vittinghoff, Glidden, Shiboski & McCulloch | Regression methods in biostatistics: linear, logistic, survival and repeated measures models (2nd edition) | Springer | 2012 | T and GLM |
| Gotelli & Ellison | A Primer of Ecological Statistics (2^nd^ edition) | Sinauer | 2013 | T |
| Cobb | Introduction to design and analysis of experiments | Wiley | 2014 | T |
| Kleinbaum, Kupper, Nizam & Rosenberg | Applied regression analysis and other multivariable methods (5^th^ edition) | Cengage Learning | 2014 | T |
| Faraway | Linear Models with R (2^nd^ edition) | CRC press | 2015 | T |
| Lawson | Design and Analysis of experiments | CRC press | 2015 | T |
| Welham, Gezan, Clark & Mead | Statistical Methods in Biology: Design and analysis of experiments and regression | CRC press | 2015 | T and GLM |
| West, Welch & Galecki | Linear Mixed Models: a practical guide using statistical software (2^nd^ edition) | CRC press | 2015 | GLM |
| Bilder & Loughin | Analysis of categorical data with R | CRC press | 2015 | GLM |
| Rosner | Fundamentals of biostatistics, (8^th^ edition) | Brooks/Cole Cengage Learning | 2016 | T |

**Appendix 2**

**Review of the procedure used with analyses of data with a non-normal error structures (counts and proportions) in articles published in peer-reviewed journals between 1980 and 2017.**

**Table 2.**  Overview of the procedures applied in the analysis of non-normal data in research published between 1980 and 2017. The articles were found by using the keyword “count” or “count data” while searching journals in the fields of biology and ecology.

| **Authors** | **Title** | **Year** | **Journal** | **Data type** | **Procedure** |
| --- | --- | --- | --- | --- | --- |
| Goulder | Seasonal variation in heterotrophic activity and population density of planktonic bacteria in a clean river | 1980 | Journal of Ecology | Count, proportion | Log and arcsine transformation |
| Capriulo and Carpenter | Grazing by 35 to 202 μm micro-zooplankton in Long Island Sound | 1980 | Marine Biology | Count | Log and square root transformation |
| Wright and Coffin | Planktonic bacteria in estuaries and coastal waters of northern Massachusetts: spatial and temporal distribution | 1983 | Marine Ecology Progress series | Count | Natural log transformation |
| Broom | Mortality and production in natural, artificially-seeded and experimental populations of *Anadara granosa* (Bivalvia: Arcidae) | 1983 | Oecologia | Count | Natural log transformation |
| Fuentes et al. | Shrub clumps of the chilean matorral vegetation: Structure and possible maintenance mechanisms | 1984 | Oecologia | Proportion | Arcsine transformation |
| Fox | Incidence of dioecy in relation to growth form, pollination and dispersal | 1985 | Oecologia | Proportion | Arcsine transformation |
| Thomson | Pollen transport and deposition by bumble bees in *Erythronium*: influences of floral nectar and bee grooming | 1986 | Journal of Ecology | Count | Natural log(*y*+1) transformation |
| Lacy and Bock | The correlation between range size and local abundance of some North American birds | 1986 | Ecology | Counts | Log transformation |
| Soballe and Kimmel | A large-scale comparison of factors influencing phytoplankton abundance in rivers, lakes, and impoundments | 1987 | Ecology | Counts | Log transformation |
| Robertson | Abundances of surgeonfishes on patch-reefs in Caribbean Panama: due to settlement, or post-settlement events? | 1988 | Marine Biology | Count | Arcsine transformation |
| Stoner | A nursery ground for four tropical *Penaeus* species: Laguna Joyuda, Puerto Rico | 1988 | Marine Ecology Progress series | Count | Log transformation |
| McArdle and Blackwell | Measurement of density variability in the bivalve *Chione stutchburyi* using spatial autocorrelation | 1989 | Marine Ecology Progress series | Count | Log(*y*+1) transformation |
| Rubenstein and Hohmann | Parasites and social behavior of island feral horses | 1989 | Oikos | Count | Log transformation |
| James et al. | Reproductive cycle of the brachiopod *Terebratulina retusa* on the west coast of Scotland | 1991 | Marine Biology | Proportion | Arcsine transformation |
| Fowler et al. | Effects of avian predation on grasshopper populations in North Dakota grasslands | 1991 | Ecology | Count | Log transformation |
| Schaff et al. | Spatial heterogeneity of benthos on the Carolina continental slope: large (100 km)-scale variation | 1992 | Marine Ecology Progress series | Count | Log transformation |
| Poiani | Ectoparasitism as a possible cost of social life: A comparative analysis using australian passerines (Passeriformes) | 1992 | Oecologia | Count | Log transformation |
| McCook and Chapman | Community succession following massive ice-scour on a rocky intertidal shore: recruitment, competition and predation during early, primary succession | 1993 | Marine Biology | Count, proportion | Transformation (various) |
| Rosenheim et al. | Influence of intraguild predation among generalist insect predators on the suppression of an herbivore population | 1993 | Oecologia | Count | Natural log transformation |
| Fox | Size and sex allocation in monoecious woody plants | 1993 | Oecologia | Count | Log transformation |
| Lawler | Direct and indirect effects in microcosm communities of protists | 1993 | Oecologia | Count | Log transformation |
| Obeso and Grubb | Iinteractive effects of extent and timing of defoliation, and nutrient supply on reproduction in a chemically protected annual *Senecio vulgaris* | 1994 | Oikos | Count | Square root transformation |
| Murray and Dickman | Granivory and microhabitat use in australian desert rodents: Are seeds important? | 1994 | Oecologia | Proportion | Arcsine transformation |
| Heard | Pitcher-plant midges and mosquitoes: a processing chain commensalism | 1994 | Ecology | Counts | Log transformation |
| Lochmiller et al. | Temporal variation in humoral and cell-mediated immune response in a *Sigmodon hispidus* population | 1994 | Ecology | Counts | Log transformation |
| Haddon and Willis | Morphometric and meristic comparison of orange roughy (*Hoplostethus atlanticus:* Trachichthyidae) from the Puysegur Bank and Lord Howe Rise, New Zealand, and its implications for stock structure | 1995 | Marine Biology | Count | Log transformation |
| McCormick | Fish feeding on mobile benthic invertebrates: influence of spatial variability in habitat associations | 1995 | Marine Biology | Count | Log transformation |
| Walters and Wethey | Settlement and early post-settlement survival of sessile marine invertebrates on topographically complex surfaces: the importance of refuge dimensions and adult morphology | 1996 | Marine Ecology Progress series | Count, proportions | Log transformation |
| Kerdelhué and Rasplus | Non-pollinating afrotropical fig wasps affect the fig-pollinator mutualism in *Ficus* within the subgenus *Sycomorus* | 1996 | Oikos | Count | Log transformation |
| Kitajima and Tilman | Seed banks and seedling establishment on an experimental productivity gradient | 1996 | Oikos | Count | Log transformation |
| Gomez et al. | Experimental study of pollination by ants in mediterranean high mountain and arid habitats | 1996 | Oecologia | Count and proportion | Arcsine transformation |
| Meekan and Choat | Latitudinal variation in abundance of herbivorous fishes: a comparison of temperate and tropical reefs | 1997 | Marine Biology | Count | Log transformation |
| Terborgh et al. | Bird communities in transition: The Lago Guri islands | 1997 | Ecology | Count | Square root transformation |
| Schmiegelow et al. | Are boreal birds resilient to forest fragmentation? An experimental study of short-term community responses | 1997 | Ecology | Count + proportion | Log transformation |
| Gillanders and Kingsford | Influence of habitat on abundance and size structure of a large temperate-reef fish, *Achoerodus viridis* (Pisces: Labridae) | 1998 | Marine Biology | Count | Double square root transformation |
| Evans and Geffen | Male characteristics, sperm traits, and reproductive success in winter-spawning Celtic Sea Atlantic herring, *Cluepea harengus* | 1998 | Marine Biology | Proportions | Arcsine transformation |
| Christe et al. | Immunocompetence and nestling survival in the house martin: The tasty chick hypothesis | 1998 | Oikos | Count | Log transformation |
| Ots and Hõrak | Health impact of blood parasites in breeding great tits | 1998 | Oecologia | Count and proportion | Log transformation |
| Watson et al. | Population dynamics of scottish rock ptarmigan cycles | 1998 | Ecology | Count | Square root and log transformation |
| Cochran-Stafira and von Ende | Integrating bacteria into food webs: Studies with *Sarracenia purpurea* inquilines | 1998 | Ecology | Counts | Log transformation |
| Rank et al. | Host preference and larval performance of the salicylate-using leaf beetle *Phratora vitellinae* | 1998 | Ecology | Count + proportion | Log transformation |
| Sieving and Willson | Nest predation and avian species diversity in Northwestern forest understory | 1998 | Ecology | Proportion | Transformation |
| Gaston et al. | The anatomy of the interspecific abundance-range size relationship for the British avifauna: I. Spatial patterns | 1998 | Ecology Letters | Counts | Log transformation |
| Pollard and Greatorex-Davies | Increased abundance of the red admiral butterfly *Vanessa atalanta* in Britain: The roles of immigration, overwintering and breeding within the country | 1998 | Ecology Letters | Counts | Log transformation |
| Sternberg et al. | Plant community dynamics in a calcareous grassland under climate change manipulations | 1999 | Plant Ecology | Count | Arcsine and square root transformations |
| Pfab and Witkowski | Contrasting effects of herbivory on plant size and reproductive performance in two populations of the critically endangered species, *Euphorbia clivicola* (R. A. Dyer) | 1999 | Plant Ecology | Count | Log transformation |
| Englund | Effects of fish on the local abundance of crayfish in stream pools | 1999 | Oikos | Count | Log transformation |
| Hõrak et al. | Immune function and survival of great tit nestlings in relation to growth conditions | 1999 | Oecologia | Count and proportion | Log transformation |
| Spencer et al. | Species richness and the proportion of predatory animal species in temporary freshwater pools: Relationships with habitat size and permanence | 1999 | Ecology Letters | Proportion | Arcsine square root transformed |
| Watkinson et al. | Population dynamics of *Vulpia ciliata*: regional, patch and local dynamics | 2000 | Journal of Ecology | Count | Log transformation |
| Ortiz-Pulido and Rico-Gray | The effect of spatio-temporal variation in understanding the fruit crop size hypothesis | 2000 | Oikos | Count | GLM |
| Strong et al. | Bird predation on herbivorous insects: Indirect effects on sugar maple saplings | 2000 | Oecologia | Proportion | Arcsine transformation |
| Raymond et al. | Intraguild predators and the spatial distribution of a parasitoid | 2000 | Oecologia | Count | Log transformation |
| Eom et al. | Host plant species effects on arbuscular mycorrhizal fungal communities in tallgrass prairie | 2000 | Oecologia | Count | Rank transformation |
| Grosholz et al. | The impacts of a nonindigenous marine predator in a California bay | 2000 | Ecology | Count + proportion | Arcsine, square root, and log transformation |
| Lindenmayer et al. | A simple landscape-scale test of a spatially explicit population model: Patch occupancy in fragmented South-Eastern Australian forests | 2001 | Oikos | Count | Logistic regression |
| Pöysä | Dynamics of habitat distribution in breeding mallards: Assessing the applicability of current habitat selection models | 2001 | Oikos | Count | Log and arcsine transformation |
| Dejean | Ant protection of a heteropteran trophobiont against a parasitoid Wasp | 2001 | Oecologia | Count | GLM |
| Dempsteret al. | Attraction of wild fish to sea-cage fish farms in the south-western Mediterranean Sea: spatial and short-term temporal variability | 2002 | Marine Ecology Progress series | Count | Natural log(*y*+1) and square root transformation |
| Kendall et al. | Influence of male size and mating history on sperm content of ejaculates of the blue crab *Callinectes sapidus* | 2002 | Marine Ecology Progress series | Count | Log transformation |
| Skov et al. | Quantifying the density of mangrove crabs: Ocypodidae and Grapsidae | 2002 | Marine Biology | Count, proportions | Arcsine transformation |
| Hutchings et al. | Grazing decisions of soay sheep, ovis aries, on St Kilda: A consequence of parasite distribution? | 2002 | Oikos | Count | GLMM |
| Comes et al. | Are differences in seed mass among species important in structuring plant communities? Evidence from analyses of spatial and temporal variation in dune-annual populations | 2002 | Oikos | Count | GLM |
| Shears and Babcock | Marine reserves demonstrate top-down control of community structure on temperate reefs | 2002 | Oecologia | Count | GLM |
| Montiel and Montana | Seed bank dynamics of the desert cactus *Opuntia rastrera* in two habitats from the Chihuahuan desert | 2003 | Plant Ecology | Count | Arcsine transformation |
| Dukas and Morse | Crab spiders affect flower visitation by bees | 2003 | Oikos | Count | Log transformation |
| Pöysä and Pesonen | Density dependence, regulation and open-closed populations: Insights from the wigeon, *Anas penelope* | 2003 | Oikos | Count | Log transformation |
| Saks et al. | Carotenoid-based plumage coloration of male greenfinches reflects health and immunocompetence | 2003 | Oecologia | Count | Log transformation |
| Rutledge et al. | Effects of a simple plant morphological mutation on the arthropod community and the impacts of predators on a principal insect herbivore | 2003 | Oecologia | Count | Log transformation |
| Vorburger et al. | Explaining the coexistence of asexuals with their sexual progenitors: No evidence for general-purpose genotypes in obligate parthenogens of the peach-potato aphid, *Myzus persicae* | 2003 | Ecology Letters | Counts | Square root transformation |
| Ogutu and Owen-Smith | ENSO, rainfall and temperature influences on extreme population declines among African savanna ungulates | 2003 | Ecology Letters | Counts | Log transformation |
| Figuerola and Green | Effects of seed ingestion and herbivory by waterfowl on seedling establishment: a field experiment with *Wigeongrass ruppia maritima* in Doñana, South-West Spain | 2004 | Plant Ecology | Count | GLM |
| Forstmeier and Weiss | Adaptive plasticity in nest-site selection in response to changing predation risk | 2004 | Oikos | Count | Square root transformation |
| Bos et al. | Dark-bellied brent geese aggregate to cope with increased levels of primary production | 2004 | Oikos | Count | Square root transformation |
| Selmi and Boulinier | Distribution-abundance relationship for passerines breeding in tunisian oases: Test of the sampling hypothesis | 2004 | Oecologia | Count | Log transformation |
| Godfrey et al. | Ecological risk assessment of transgenic pasture plants: A community gradient modelling approach | 2004 | Ecology Letters | Counts | Log and square root transformation |
| Ferrari et al. | The role of host sex in parasite dynamics: field experiments on the yellow-necked mouse *Apodemus flavicollis* | 2004 | Ecology Letters | Counts | GLMM |
| Womble et al. | Distribution of Steller sea lions *Eumetopias jubatus* in relation to spring-spawning fish in SE Alaska | 2005 | Marine Ecology Progress series | Count | Square root transformation |
| Dempster et al. | Vertical variability of wild fish assemblages around sea-cage fish farms: implications for management | 2005 | Marine Ecology Progress series | Count | Log transformation |
| Beckage et al. | Survival of tree seedlings across space and time: estimates from long-term count data | 2005 | Journal of Ecology | Count | GLM |
| Holmstad et al. | The influence of a parasite community on the dynamics of a host population: A longitudinal study on willow ptarmigan and their parasites | 2005 | Oikos | Count | GLM |
| Lavergne et al. | Limitations on reproductive success in endemic *Aquilegia viscosa* (Ranunculaceae) relative to its widespread congener *Aquilegia vulgaris*: The interplay of herbivory and pollination | 2005 | Oecologia | Count | GLM |
| Mougeot et al. | Interactions between population processes in a cyclic species: parasites reduce autumn territorial behaviour of male red grouse | 2005 | Oecologia | Count | GLM |
| Mulder et al. | Bacterial traits, organism mass, and numerical abundance in the detrital soil food web of Dutch agricultural grasslands | 2005 | Ecology Letters | Counts | GLM |
| Kunert et al. | Alarm pheromone mediates production of winged dispersal morphs in aphids | 2005 | Ecology Letters | Counts | GLM |
| Klemola et al. | Geographically partitioned spatial synchrony among cyclic moth populations | 2006 | Oikos | Count | Log transformation |
| Liess and Diehl | Effects of enrichment on protist abundances and bacterial composition in simple microbial communities | 2006 | Oikos | Count | Log (x+1) transformation |
| Julliard et al. | Spatial segregation of specialists and generalists in bird communities | 2006 | Ecology Letters | Counts | GLM |
| Redpath et al. | Testing the role of parasites in driving the cyclic population dynamics of a gamebird | 2006 | Ecology Letters | Counts | Log transformation |
| Jiguet et al. | Thermal range predicts bird population resilience to extreme high temperatures | 2006 | Ecology Letters | Counts | GLM |
| Walsh et al. | Maladaptive changes in multiple traits caused by fishing: impediments to population recovery | 2006 | Ecology Letters | Counts | Arcsine transformation |
| Hammerstrom et al. | Response and recovery dynamics of seagrasses *Thalassia testudinum* and *Syringodium filiforme* and macroalgae in experimental motor vessel disturbances | 2007 | Marine Ecology Progress series | Count | Square root transformation |
| Creed and De Paula | Substratum preference during recruitment of two invasive alien corals onto shallow-subtidal tropical rocky shores | 2007 | Marine Ecology Progress series | Count | Square root transformation |
| Spottiswoode | Phenotypic sorting in morphology and reproductive investment among sociable weaver colonies | 2007 | Oecologia | Count | GLM |
| Barriault et al. | Pollination ecology and reproductive success in Jack-in-the-pulpit (*Arisaema triphyllum*) in Québec (Canada) | 2008 | Plant Biology | Count | GLM |
| Cao and Kudo | Size-dependent sex allocation in a monocarpic perennial herb, *Cardiocrinum cordatum* (Liliaceae) | 2008 | Plant Ecology | Count | GLM and GLMM |
| Marx and Walters | Survival of tree seedlings on different species of decaying wood maintains tree distribution in Michigan hemlock-hardwood forests | 2008 | Journal of Ecology | Count | GLM |
| Devictor et al. | Distribution of specialist and generalist species along spatial gradients of habitat disturbance and fragmentation | 2008 | Oikos | Count | Log transformation |
| Reynolds and Fenster | Point and interval estimation of pollinator importance: A study using pollination data of *Silene caroliniana* | 2008 | Oecologia | Count | GLM |
| Sharples et al. | Estimating seasonal abundance of a central place forager using counts and telemetry data | 2009 | Marine Ecology Progress series | Count, probabilities | GLMM |
| Gonzalez-Alday et al. | Evaluating different harvest intensities over understory plant diversity and pine seedlings, in a *Pinus pinaster* ait. natural stand of Spain | 2009 | Plant Ecology | Count, biodiversity index | Square root and arcsine transformation |
| McCay et al. | Deposition of exotic bird-dispersed seeds into three habitats of a fragmented landscape in the Northeastern United States | 2009 | Plant Ecology | Count | Square root transformation |
| Pennings et al. | Latitudinal variation in herbivore pressure in Atlantic Coast salt marshes | 2009 | Ecology | Counts | Log transformation |
| Castellanos-Galindo et al. | Tidal influences on fish distributions on tropical eastern Pacific rocky shores (Colombia) | 2010 | Marine Ecology Progress series | Count | GLM |
| Chakraborty et al. | Use of response surface methodology for optimization of a shoot regeneration protocol in *Basilicum polystachyo* | 2010 | In vitro cell dev. Biol. -Plant | Unclear | GLM |
| McCauley et al. | Acute effects of removing large fish from a near-pristine coral reef | 2010 | Marine Biology | Count and biomass | GLMM and square root transformation |
| Douglas et al. | How important are climate-induced changes in host availability for population processes in an obligate brood parasite, the European cuckoo? | 2010 | Oikos | Count | GLM |
| Anagnostou et al. | Friendly food for fitter flies? – Influence of dietary microbial species on food choice and parasitoid resistance in *Drosophila* | 2010 | Oikos | Count | GLM |
| Gilbert | Altitudinal patterns of tick and host abundance: A potential role for climate change in regulating tick-borne diseases? | 2010 | Oecologia | Count | GLMM |
| Bonebrake and Beissinger | Predation and infanticide influence ideal free choice by a parrot occupying heterogeneous tropical habitats | 2010 | Oecologia | Count | Arcsine and log (x+1) transformation |
| Hoover-Miller et al. | Persistent decline in abundance of harbor seals *Phoca vitulina richardsi* over three decades in Aialik Bay, an Alaskan tidewater glacial fjord | 2011 | Marine Ecology Progress series | Count | GLM |
| Greene et al. | Grazing impact of the invasive clam *Corbula amurensis* on the microplankton assemblage of the northern San Francisco Estuary | 2011 | Marine Ecology Progress series | Biomass | GLM |
| Hinz et al. | Effects of scallop dredging on temperate reef fauna | 2011 | Marine Ecology Progress series | Count | GLM |
| Danielson et al. | Chemical and transcriptional responses of Norway spruce genotypes with different susceptibility to *Heterobasidion* spp. infection | 2011 | Plant Biology | Counts | Log transformation |
| Thébault et al. | Polyploidy and invasion success: trait trade-offs in native and introduced cytotypes of two Asteraceae species | 2011 | Plant Ecology | Count | GLM |
| Michalet et al. | Phenotypic variation in nurse traits and community feedbacks define an alpine community | 2011 | Ecology Letters | Counts | Transformation (unspecified) |
| Sveegaard at al. | Spatial interactions between marine predators and their prey: herring abundance as a driver for the distributions of mackerel and harbour porpoise | 2012 | Marine Ecology Progress series | Count | GLM |
| Turtureanu and Dengler | Different aspects of plant diversity show contrasting patterns in Carpathian forest openings | 2012 | Plant Ecology | Count | GLM |
| Hurst et al. | Thermal reaction norms for growth vary among cohorts of Pacific cod (*Gadus macrocephalus*) | 2012 | Marine Biology | Counts | Natural log transformation |
| Mongomery and Rathcke | Effects of floral restrictiveness and stigma size on heterospecific pollen receipt in a prairie community | 2012 | Oecologia | Count | Log transformation |
| Bell et al. | Putting the brakes on a cycle: Bottom-up effects damp cycle amplitude | 2012 | Ecology Letters | Counts | GLM/GAM |
| Patot et al. | An inherited virus influences the coexistence of parasitoid species through behaviour manipulation | 2012 | Ecology Letters | Counts | Square root transformation |
| Langwig et al. | Sociality, density-dependence and microclimates determine the persistence of populations suffering from a novel fungal disease, white-nose syndrome | 2012 | Ecology Letters | Counts | GLM and log transformation |
| Bracken and Low | Realistic losses of rare species disproportionately impact higher trophic levels | 2012 | Ecology Letters | Counts | GLM |
| Cox et al. | Combined spatial and tidal processes identify links between pelagic prey species and seabirds | 2013 | Marine Ecology Progress series | Dispersion coefficient | Log transformation |
| Andresen et al. | Growth and size-dependent loss of newly settled bivalves in two distant regions of the Wadden Sea | 2013 | Marine Ecology Progress series | Count | GLM |
| Neuenkamp et al. | Impact of management on biodiversity-biomass relations in Estonian flooded meadows | 2013 | Plant Ecology | Count | GLM |
| Goring et al. | Pollen assemblage richness does not reflect regional plant species richness: a cautionary tale | 2013 | Journal of Ecology | Counts | GLM |
| Sundell et al. | Numerical response of small mustelids to vole abundance: delayed or not? | 2013 | Oikos | Count | Log transformation |
| Stevens and Dennis | Wildlife mortality from infrastructure collisions: Statistical modeling of count data from carcass surveys | 2013 | Ecology | Count and proportion | GLM |
| Maas et al. | Bats and birds increase crop yield in tropical agroforestry landscapes | 2013 | Ecology Letters | Counts | Log transformation |
| Allington et al. | Niche opportunities and invasion dynamics in a desert annual community | 2013 | Ecology Letters | Counts | GLMM |
| Hughes et al. | Changes in the spatial distribution of spawning activity by north-east Atlantic mackerel in warming seas: 1977-2010 | 2014 | Marine Biology | Count | GLM |
| Zhang et al. | Proteogenomic characterization of human colon and rectal cancer | 2014 | Nature | Counts | Log transformation |
| Kessler et al. | Species richness - productivity relationships of tropical terrestrial ferns at regional and local scales | 2014 | Journal of Ecology | Counts | Log transformation |
| Zimmerman and Cardinale | Is the relationship between algal diversity and biomass in North American lakes consistent with biodiversity experiments? | 2014 | Oikos | Count | Log transformation |
| Lowe et al. | Broad sampling and diverse biomarkers allow characterization of nearshore particulate organic matter | 2014 | Oikos | Count | Arcsine transformation |
| Bertness et al. | Experimental predator removal causes rapid salt marsh die-off | 2014 | Ecology Letters | Counts | Transformation (unspecified) |
| Jones et al. | Patterns of space use in sympatric marine colonial predators reveal scales of spatial partitioning | 2015 | Marine Ecology Progress series | Count | GLM |
| Loy et al. | Asteraceae invaders have limited impacts on the pollination of common native annual species in SW Western Australia’s open woodland wildflower communities | 2015 | Plant Ecology | Count, proportions | GLMM |
| Crowther et al. | Mapping tree density at a global scale | 2015 | Nature | Count | GLM |
| Cox et al. | Effects of ocean acidification on *Posidonia oceanica* epiphytic community and shoot productivity | 2015 | Journal of Ecology | Count, proportions | Transformation (unspecified) |
| Kellermann and Van Riper III | Detecting mismatches of bird migration stopover and tree phenology in response to changing climate | 2015 | Oecologia | Count and proportion | GLM |
| Brown et al. | Disentangling legacy effects from environmental ﬁlters of postﬁre assembly of boreal tree assemblages | 2015 | Ecology | Count | GLM |
| Doody et al. | Invasive toads shift predator–prey densities in animal communities by removing top predators | 2015 | Ecology | Count | GLM |
| Painter et al. | Recovering aspen follow changing elk dynamics in Yellowstone: evidence of a trophic cascade | 2015 | Ecology | Counts | Log transformation |
| Lu et al. | Climate warming increases biological control agent impact on a non-target species | 2015 | Ecology Letters | Counts | Square root transformation |
| Knauer and Schiestl | Bees use honest ﬂoral signals as indicators of reward when visiting ﬂowers | 2015 | Ecology Letters | Counts | Log transformation |
| Vijendravarma et al. | Gut physiology mediates a trade-off between adaptation to malnutrition and susceptibility to food-borne pathogens | 2015 | Ecology Letters | Counts and proportion | Square root and log transformation |
| Pesendorfer and Koenig | The effect of within‑year variation in acorn crop size on seed harvesting by avian hoarders | 2016 | Oecologia | Count and proportion | GLM |
| Crone and Williams | Bumble bee colony dynamics: Quantifying the importance of land use and ﬂoral resources for colony growth and queen production | 2016 | Ecology Letters | Counts | GLM/GLMM |
| Ramiro et al. | Facilitation through altered resource availability in a mixed-species rodent malaria infection | 2016 | Ecology Letters | Counts | Log transformation |
| Thomson | Local bumble bee decline linked to recovery of honey bees, drought effects on ﬂoral resources | 2016 | Ecology Letters | Counts | Log transformation |
| Mooney et al. | Abiotic mediation of a mutualism drives herbivore abundance | 2016 | Ecology Letters | Counts | Log transformation |
| Doody et al. | Chronic effects of an invasive species on an animal community | 2017 | Ecology | Counts | GLM |

**Appendix 3**

Number of train-to-train collisions in relation to the year, from Agresti (1996), page 83.

- “Tcrash” refers to the number of train crashes (response variable).
- “Year” refers to the year (explanatory variable, continuous).

**Square root transformation**

Model:

modelCarT2<-lm((sqrt(Tcrash))~Year, data=dataTrain)


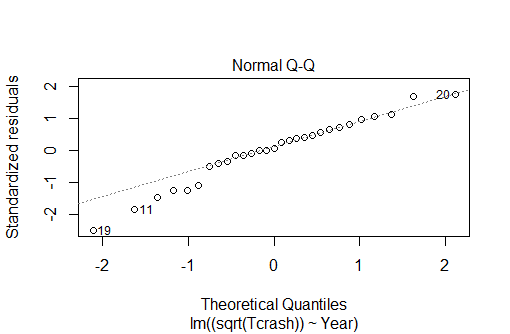

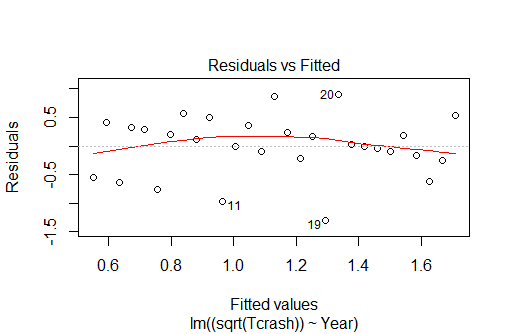
Graphical assessment of the assumptions:

ANOVA table:

| **Source of variation** | **Sum Sq** | **Df** | **F value** | **Pr(>F)** |
| --- | --- | --- | --- | --- |
| Year | 3.4784 | 1 | 12.501 | 0.00149 |
| Residuals | 7.5128 | 27 |  |  |

Coefficients:

|  | **Estimate** | **Pr(>\|t\|)** | **Coefficient** |
| --- | --- | --- | --- |
| (Intercept) | 83.46369 | 0.00131 | 6966.188 |
| Year | -0.04139 | 0.00149 | -0.001713 |

**Logarithmic transformation**

Model:

modelCarT1<-lm((log(Tcrash+1))~Year, data=dataTrain)


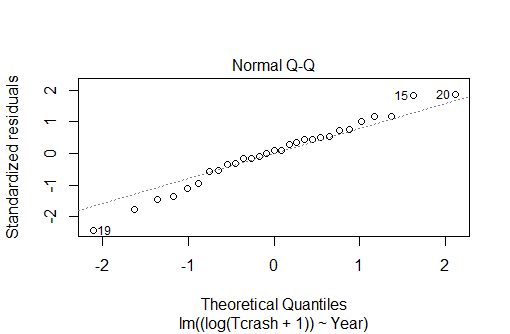

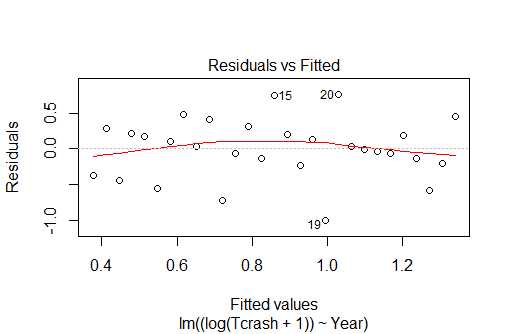
Graphical assessment of the assumptions:

ANOVA table:

| **Source of variation** | **Sum Sq** | **Df** | **F value** | **Pr(>F)** |
| --- | --- | --- | --- | --- |
| **Year** | 2.4073 | 1 | 13.705 | 0.0009681 |
| **Residuals** | 4.7425 | 27 |  |  |

Coefficients:

|  | **Estimate** | **Pr(>\|t\|)** | **Coefficient** |
| --- | --- | --- | --- |
| **(Intercept)** | 69.352533 | 0.000858 | 1.32E+30 |
| **Year** | -0.034436 | 0.000968 | -0.03385 |

**Generalised linear model (Poisson distribution)**

Model:

modelCarG<-glm(Tcrash~Year, family=poisson, data=dataTrain)


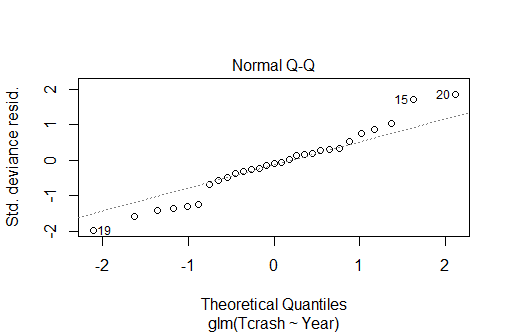

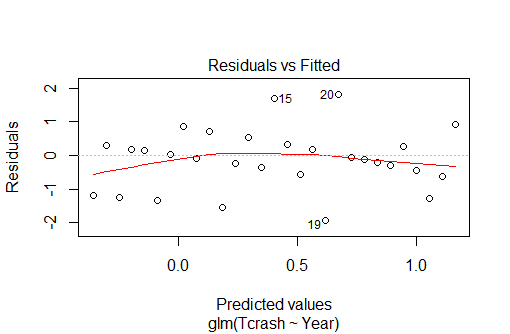
Graphical assessment of the assumptions:

ANOVA table:

|  | **LR Chisq** | **Df** | **Pr(>Chisq)** |
| --- | --- | --- | --- |
| **Year** | 9.2954 | 1 | 0.002297 |

Coefficients:

|  | **Estimate** | **Pr(>\|t\|)** | **Coefficient** |
| --- | --- | --- | --- |
| **(Intercept)** | 108.19335 | 0.00292 | 9.72E+46 |
| **Year** | -0.05419 | 0.00308 | -0.05275 |

**Summary of Appendix 3**

- Residuals: the residual vs fit plots obtained for each of the three models seem equivalent and acceptable.
- Outcome of the p-value: for all three models, the decision based on the p-value does not change, as we observe a significant p-value for the regression in each of the models.
- Estimated coefficients:

Comparison between the three models:

|  | **Square root** | **Log(*y*+1)** | **GLM** |
| --- | --- | --- | --- |
| **Intercept** | 6966.188 | 1.32E+30 | 9.72E+46 |
| **Year** | -0.001713 | -0.03385 | -0.05275 |

The table above shows that both models using transformations underestimate the values of estimate and intercept. The model with the square root transformation yields the largest bias to the most accurate value of the estimate (which is obtained from the generalised linear model – Stroup 2012).

**Appendix 4**

Number of train-to-train collisions in relation to the distance travelled by the trains (train-kilometers), from Agresti (1996), page 83.

- “Tcrash” refers to the number of train crashes (response variable).
- “Tkm” refers to the train-kilometers (explanatory variable, continuous).

**Square root transformation**

Model:

modelCarT2<-lm((sqrt(Tcrash))~TKm, data=dataTrain)


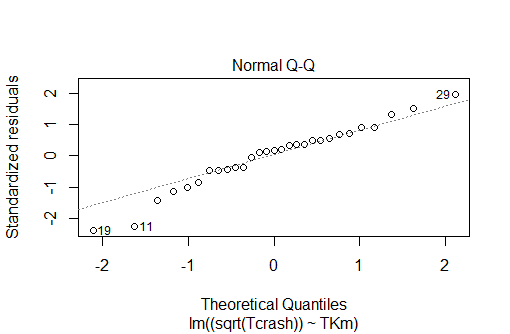

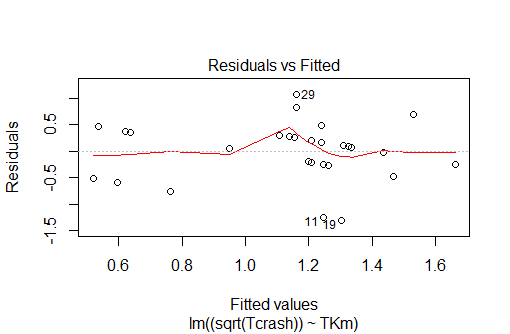
Graphical assessment of the assumptions:

ANOVA table:

| **Source of variation** | **Sum Sq** | **Df** | **F value** | **Pr(>F)** |
| --- | --- | --- | --- | --- |
| **TKm** | 2.5637 | 1 | 8.2136 | 0.00796 |
| **Residuals** | 8.4276 | 27 |  |  |

Coefficients:

|  | **Estimate** | **Pr(>\|t\|)** | **Coefficient** |
| --- | --- | --- | --- |
| **(Intercept)** | 4.571928 | 0.000765 | 20.90253 |
| **TKm** | -0.007823 | 0.007960 | -6.12E-05 |

**Logarithmic transformation**

Model:

modelCarT1<-lm((log(Tcrash+1))~TKm, data=dataTrain)


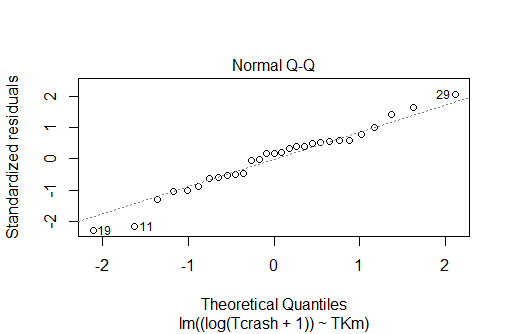

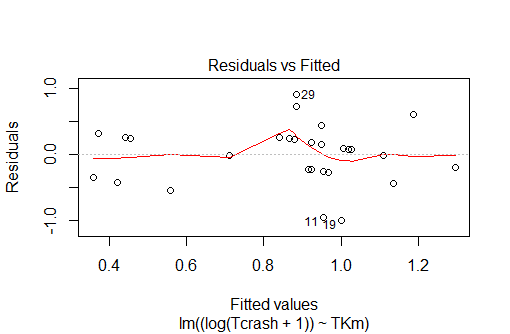
Graphical assessment of the assumptions:

ANOVA table:

| **Source of variation** | **Sum Sq** | **Df** | **F value** | **Pr(>F)** |
| --- | --- | --- | --- | --- |
| **TKm** | 1.7340 | 1 | 8.6445 | 0.00665 |
| **Residuals** | 5.4158 | 27 |  |  |

Coefficients:

|  | **Estimate** | **Pr(>\|t\|)** | **Coefficient** |
| --- | --- | --- | --- |
| **(Intercept)** | 3.689628 | 0.000715 | 39.02995 |
| **TKm** | -0.006434 | 0.006650 | -0.00641 |

**Generalised linear model (Poisson distribution)**

Model:

modelCarG<-glm(Tcrash~TKm, family=poisson, data=dataTrain)


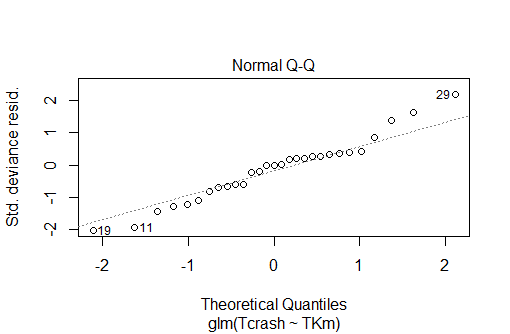

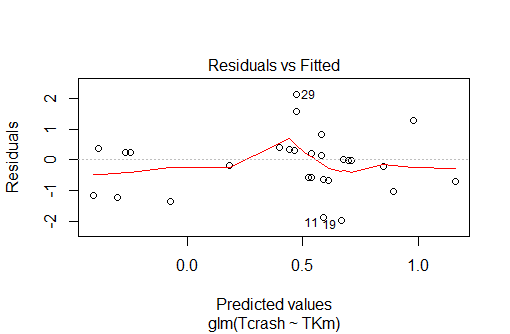
Graphical assessment of the assumptions:

ANOVA table:

| **Source of variation** | **LR Chisq** | **Df** | **Pr(>Chisq)** |
| --- | --- | --- | --- |
| **TKm** | 6.3958 | 1 | 0.01144 |

Coefficients:

|  | **Estimate** | **Pr(>\|z\|)** | **Coefficient** |
| --- | --- | --- | --- |
| **(Intercept)** | 5.15436 | 0.00758 | 1.73E+02 |
| **TKm** | -0.01074 | 0.01725 | -0.010682532 |

**Summary of Appendix 4**

- Residuals: the residual vs fit plots obtained for each of the three models seem equivalent but not optimal. The data seems to disperse slightly in the center. In the case of the generalised linear model, the negative binomial distribution did not yield better results.
- Outcome of the p-value: although the values fluctuate, the decision on the p-value remains the same will all three models, being significant in all cases.
- Estimates of the coefficients:

Comparison between the three models:

|  | **Square root** | **Log(*y*+1)** | **GLM** |
| --- | --- | --- | --- |
| **Intercept** | 20.90253 | 39.02995 | 1.73E+02 |
| **TKm** | -0.0000612 | -0.00641 | -0.010682532 |

The table above shows that both models using transformations underestimate the values of the estimate and intercept. The model with the square root transformation yields the largest bias to the true value of the estimate (which is obtained from the generalised linear model – Stroup 2012).

**Appendix 5**

Number of train-to-car collisions in relation to the distance travelled by the trains (train-kilometers), from Agresti (1996), page 83.

- “Ccrash” refers to the number of crashes between trains and cars (response variable)
- “Tkm” refers to the train-kilometers (explanatory variable, continuous).

**Square root transformation**

Model:

modelCarT2<-lm((sqrt(Ccrash))~TKm, data=dataTrain)


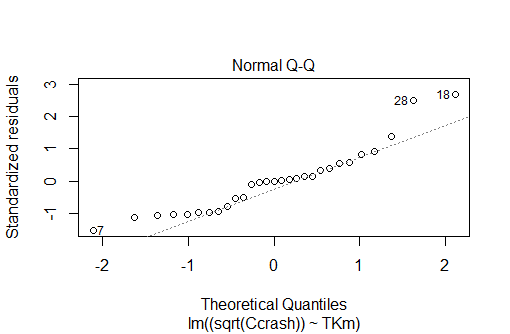

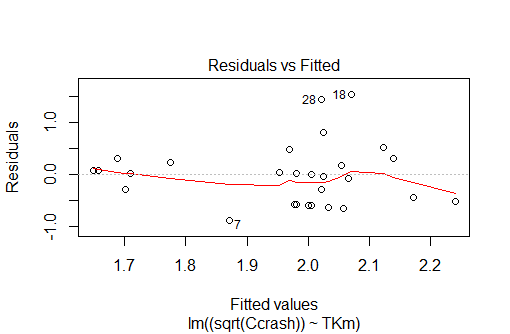
Graphical assessment of the assumptions:

ANOVA table:

| **Source of variation** | **Sum Sq** | **Df** | **F value** | **Pr(>F)** |
| --- | --- | --- | --- | --- |
| **TKm** | 0.6879 | 1 | 1.9773 | 0.1711 |
| **Residuals** | 9.3929 | 27 |  |  |

Coefficients:

|  | **Estimate** | **Pr(>\|t\|)** | **Coefficient** |
| --- | --- | --- | --- |
| **(Intercept)** | 3.747547 | 0.00658 | 14.04410852 |
| **TKm** | -0.004052 | 0.17108 | 1.64187E-05 |

**Logarithmic transformation**

Model:

modelCarT1<-lm((log(Ccrash+1))~TKm, data=dataTrain)


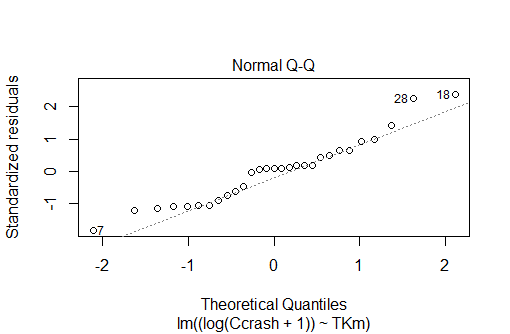

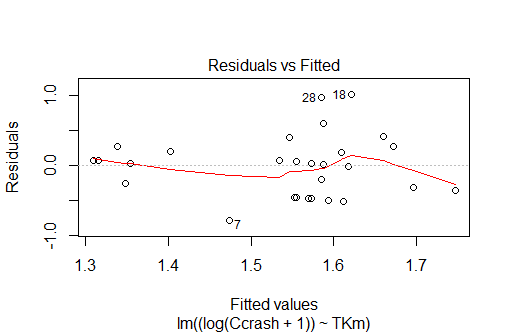
Graphical assessment of the assumptions:

ANOVA table:

| **Source of variation** | **Sum Sq** | **Df** | **F value** | **Pr(>F)** |
| --- | --- | --- | --- | --- |
| **TKm** | 0.3755 | 1 | 1.9276 | 0.1764 |
| **Residuals** | 5.2598 | 27 |  |  |

Coefficients:

|  | **Estimate** | **Pr(>\|t\|)** | **Coefficient** |
| --- | --- | --- | --- |
| **(Intercept)** | 2.860207 | 0.0057 | 16.46514 |
| **TKm** | -0.002994 | 0.1764 | -0.00299 |

**Generalised linear model (Poisson distribution)**

Model:

modelCarG<-glm(Ccrash~TKm, family=poisson, data=dataTrain)


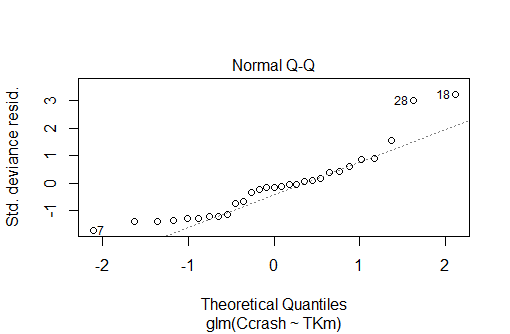

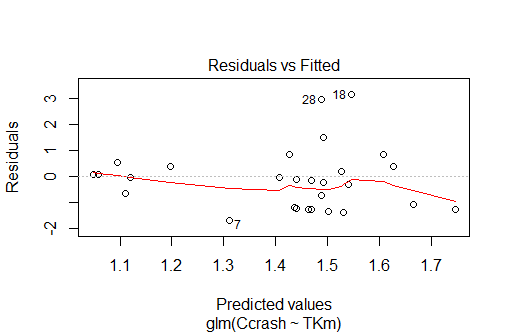
Graphical assessment of the assumptions:

ANOVA table:

| **Source of variation** | **LR Chisq** | **Df** | **Pr(>Chisq)** |
| --- | --- | --- | --- |
| **TKm** | 3.6545 | 1 | 0.05592 |

Coefficients:

|  | **Estimate** | **Pr(>\|z\|)** | **Coefficient** |
| --- | --- | --- | --- |
| **(Intercept)** | 3.524000 | 0.00159 | 33.91984 |
| **TKm** | -0.004780 | 0.06245 | -0.00477 |

**Summary of Appendix 5**

- Residuals: the residual vs fit plots obtained for each of the three models seem equivalent but not optimal. The data seems to disperse slightly in the center. In the case of the generalised linear model, the negative binomial distribution did not yield better results.
- Outcome of the p-value: the p-value is equally non-significant for the two transformation models (square root and log). The p-value obtained in the generalised linear model is also not significant, but very close to α=0.05, therefore in this case a randomisation test would be recommended.
- Estimates of the coefficients:

Comparison between the three models:

|  | **Square root** | **Log(*y*+1)** | **GLM** |
| --- | --- | --- | --- |
| **Intercept** | 14.04410852 | 16.46514 | 3.39E+01 |
| **TKm** | 1.64187E-05 | -0.00299 | -0.00477 |

The table above show that both models using transformations underestimate the values of the estimate and intercept. The model with the square root transformation yields the largest bias to the true value of the estimate (which is obtained from the generalised linear model – Stroup 2012).

**Appendix 6**

Number of train-to-car collisions in relation to the year, from Agresti (1996), page 83.

- “Ccrash” refers to the number of crashes between trains and cars (response variable)
- “Year” refers to the year (explanatory variable, continuous).

**Square root transformation**

Model:

modelCarT2<-lm((sqrt(Ccrash))~Year, data=dataTrain)


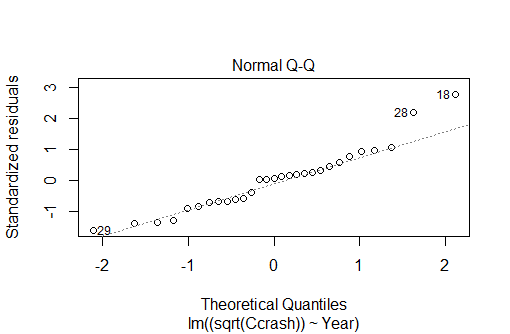

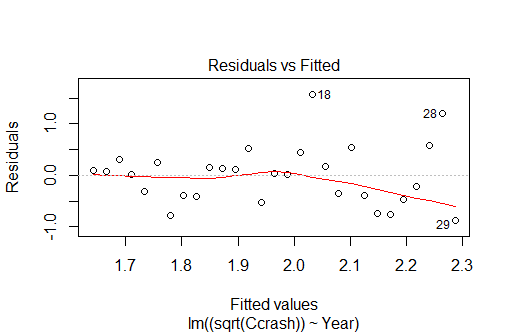
Graphical assessment of the assumptions:

ANOVA table:

| **Source of variation** | **Sum Sq** | **Df** | **F value** | **Pr(>F)** |
| --- | --- | --- | --- | --- |
| **Year** | 1.0788 | 1 | 3.2356 | 0.08324 |
| **Residuals** | 9.0020 | 27 |  |  |

Coefficients:

|  | **Estimate** | **Pr(>\|t\|)** | **Coefficient** |
| --- | --- | --- | --- |
| **(Intercept)** | 47.81592 | 0.0715 | 2286.362205 |
| **Year** | -0.02305 | 0.0832 | -0.000531303 |

**Logarithmic transformation**

Model:

modelCarT1<-lm((log(Ccrash+1))~Year, data=dataTrain)


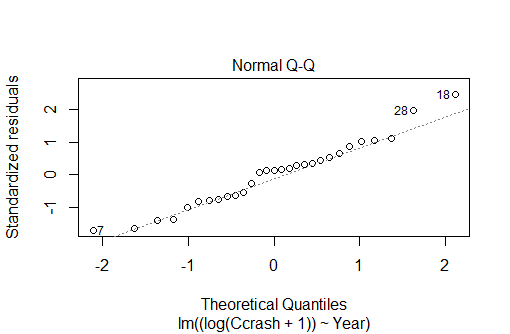

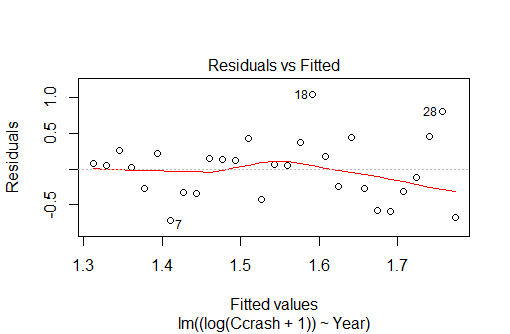
Graphical assessment of the assumptions:

ANOVA table:

| **Source of variation** | **Sum Sq** | **Df** | **F value** | **Pr(>F)** |
| --- | --- | --- | --- | --- |
| **Year** | 0.5508 | 1 | 2.9248 | 0.0987 |
| **Residuals** | 5.0845 | 27 |  |  |

Coefficients:

|  | **Estimate** | **Pr(>\|t\|)** | **Coefficient** |
| --- | --- | --- | --- |
| **(Intercept)** | 34.305539 | 0.0846 | 7.91966E+14 |
| **Year** | -0.016472 | 0.0987 | -0.016337078 |

**Generalised linear model (Poisson distribution)**

Model:

modelCarG<-glm(Ccrash~Year, family=poisson, data=dataTrain)


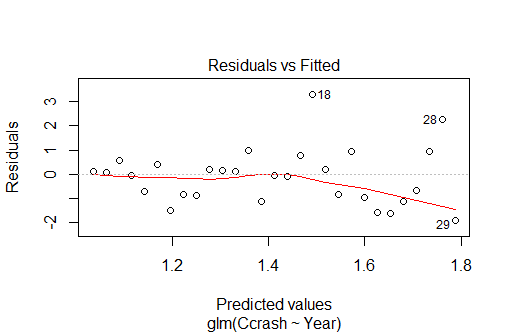

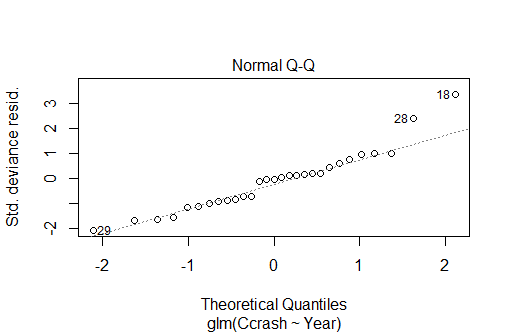
Graphical assessment of the assumptions:

ANOVA table:

|  | **LR Chisq** | **Df** | **Pr(>Chisq)** |
| --- | --- | --- | --- |
| **Year** | 6.0641 | 1 | 0.0138 |

Coefficients:

|  | **Estimate** | **Pr(>\|t\|)** | **Coefficient** |
| --- | --- | --- | --- |
| **(Intercept)** | 54.81335 | 0.0120 | 6.38E+23 |
| **Year** | -0.02685 | 0.0145 | -0.026492743 |

**Summary of Appendix 6**

- Residuals: the residual vs fit plots obtained for each of the three models seem to show the same cone shape. In the generalised linear model, using a negative binomial distribution did not improve the outcome of the residual plot. The two methods applied here (transformations and use of the generalised linear model) did not improve the fit of the model.
- Outcome of the p-value: for the two models in which a transformation was used, the decision based on the p-value does not change, as we observe a non-significant p-value in both models. However, this outcome is different from the significant p-value obtained with the generalised linear model.
- Estimates of the coefficients:

Comparison between the three models:

|  | **Square root** | **Log(*y*+1)** | **GLM** |
| --- | --- | --- | --- |
| **Intercept** | 2286.362205 | 7.91966E+14 | 6.38E+23 |
| **Year** | 0.000531303 | -0.016337078 | -0.026492743 |

The table above shows that both models using transformations underestimate the values of the estimate and intercept. The model with the square root transformation yields the largest bias to the true value of the estimate (which is obtained from the generalised linear model – Stroup 2012).

**Appendix 7**

Number of satellite horseshoe crab males relative to female carapace width, from Agresti (1996), page 76, where:

- “Satellites” refers to the number of satellites ( response variable)
- “Width” refers to the carapace width of female crabs ( explanatory variable, continuous)

**Square root transformation**

Model:

model1<-lm(sqrt.satellites~width, data=crabdata)


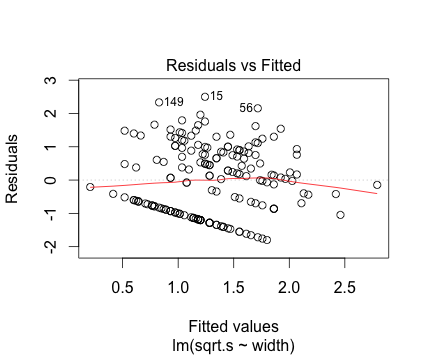

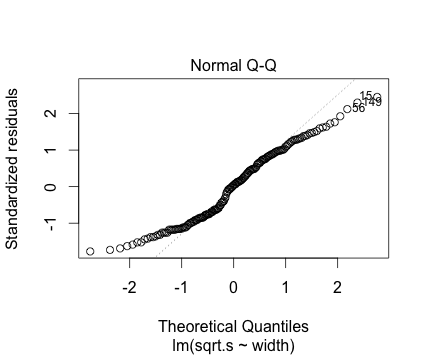
Graphical assessment of the assumptions:

ANOVA table:

| **Source of variation** | **Sum Sq** | **Df** | **F value** | **Pr(>F)** |
| --- | --- | --- | --- | --- |
| **Width** | 32.669 | 1 | 31.294 | 8.65E-08 |
| **Residuals** | 178.511 | 171 |  |  |

Coefficients:

|  | **Estimate** | **Pr(>\|t\|)** | **Coefficient** |
| --- | --- | --- | --- |
| **Intercept** | -4.13116 | -4.239 3.67e-05 | -0.390581501 |
| **Width** | 0.20664 | 5.594 8.65e-08 | 6.25997E-07 |

**Logarithmic transformation**

Model:


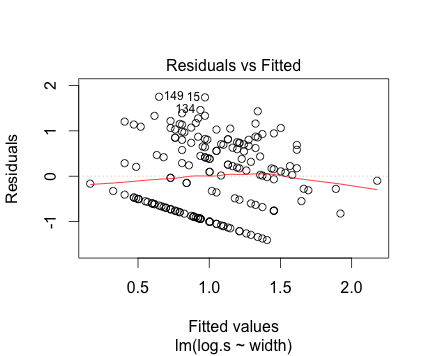
model2<-lm(log(satellites+1)~width, data=crabdata)


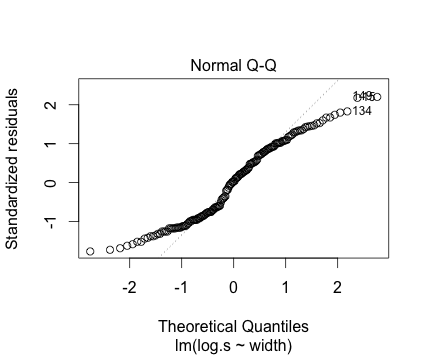
Graphical assessment of the assumptions:

ANOVA table:

| **Source of variation** | **Sum Sq** | **Df** | **F value** | **Pr(>F)** |
| --- | --- | --- | --- | --- |
| **Width** | 19.877 | 1 | 31.037 | 9.67E-08 |
| **Residuals** | 109.512 | 171 |  |  |

Coefficients:

|  | **Estimate** | **Pr(>\|t\|)** | **Coefficient** |
| --- | --- | --- | --- |
| **Intercept** | -3.22043 | 3.97E-05 | -0.960062119 |
| **Width** | 0.16118 | 9.67E-08 | 0.174896431 |

**Generalised linear model (Poisson distribution)**

Model:


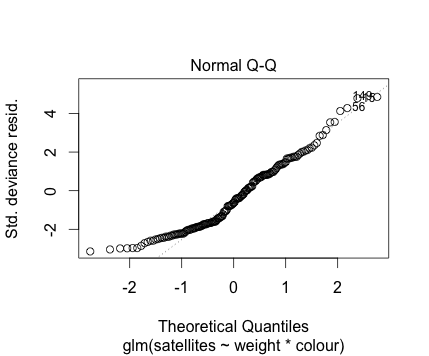

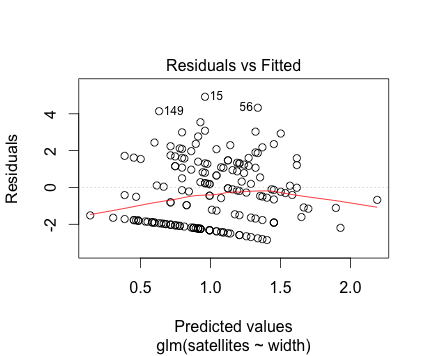
model3<-glm(satellites~width, family=poisson, data=crabdata)

Graphical assessment of the assumptions:

ANOVA table:

| **Source of variation** | **LR Chisq** | **Df** | **Pr(>Chisq)** |
| --- | --- | --- | --- |
| **Width** | 64.913 | 1 | 7.83E-16 |

Coefficients:

|  | **Estimate** | **Pr(>\|t\|)** | **Coefficient** |
| --- | --- | --- | --- |
| **Intercept** | -3.30476 | 1.10E-09 | 0.036708021 |
| **Width** | 0.16405 | < 2e-16 | 0.178273227 |

**Summary of Appendix 7**

- Residuals: the residual vs fit plots for the GLM yields a better result than those for the two transformations. The plots for both transformations show a clear fan-shape.
- Outcome of the p-value: although the value varied substantially between the linear models using transformed data and the generalised linear model, the outcome stayed the same with a significant p-value for each of the models tested.
- Estimates of the coefficients:

Comparison of the coefficients:

|  | **Square root** | **Log(*y*+1)** | **GLM** |
| --- | --- | --- | --- |
| **Intercept** | -0.390581501 | -0.960062119 | 0.036708021 |
| **Width** | 6.25997E-07 | 0.174896431 | 0.178273227 |

The table above shows that both models using transformations underestimate the values of the estimate and intercept. The square root transformation yields the largest bias to the true value of the regression coefficient, although the log transformation yields the largest bias to the true value of the intercept (which are obtained from the generalised linear model – Stroup 2012).

**Appendix 8**

Number of satellite horseshoe crab males relative to female wet weight, from Agresti (1996), page 76, where:

- “Satellites” refers to the number of satellites ( response variable)
- “Weight” refers to the wet weight of female crabs (explanatory variable, continuous)

**Square root transformation**

Model:

model1<-lm(sqrt.satellites~weight, data=crabdata)


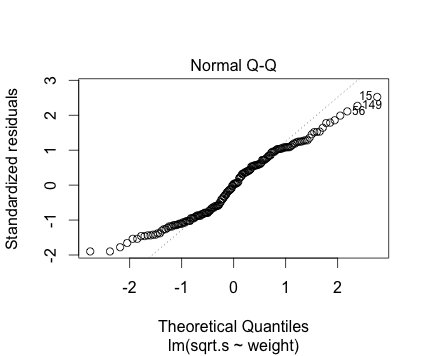

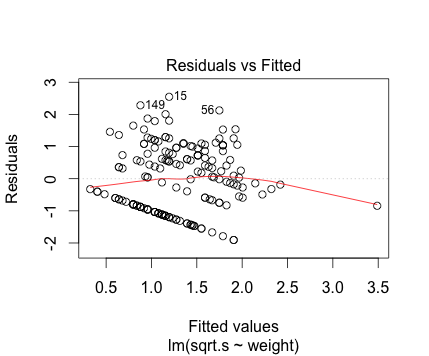
Graphical assessment of the assumptions:

ANOVA table:

| **Source of variation** | **Sum Sq** | **Df** | **F value** | **Pr(>F)** |
| --- | --- | --- | --- | --- |
| **Weight** | 35.846 | 1 | 34.959 | 1.78E-08 |
| **Residuals** | 175.334 | 171 |  |  |

Coefficients:

|  | **Estimate** | **Pr(>\|t\|)** | **Coefficient** |
| --- | --- | --- | --- |
| **Intercept** | -0.6249652 | 0.0639 | -17.06648295 |
| **Weight** | 0.0007912 | 1.78E-08 | 0.04270009 |

**Logarithmic transformation**

Model:

model2<-lm(log(satellites+1)~weight, data=crabdata)


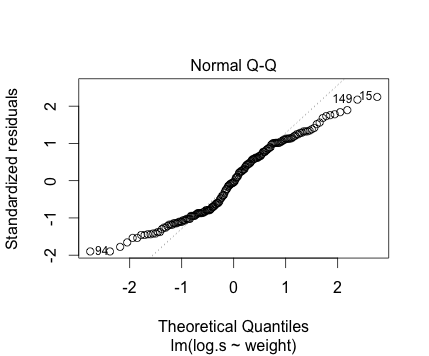

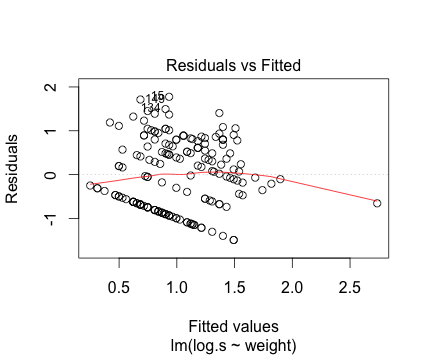
Graphical assessment of the assumptions:

ANOVA table:

| **Source of variation** | **Sum Sq** | **Df** | **F value** | **Pr(>F)** |
| --- | --- | --- | --- | --- |
| **Weight** | 22.11 | 1 | 35.243 | 1.58E-08 |
| **Residuals** | 107.28 | 171 |  |  |

Coefficients:

|  | **Estimate** | **Pr(>\|t\|)** | **Coefficient** |
| --- | --- | --- | --- |
| **Intercept** | -0.4958414 | 0.0602 | -0.39094177 |
| **Weight** | 0.0006214 | 1.58E-08 | 0.000621593 |

**Generalised linear model (Poisson distribution)**

Model:

model3<-glm(satellites~weight, family=poisson, data=crabdata)


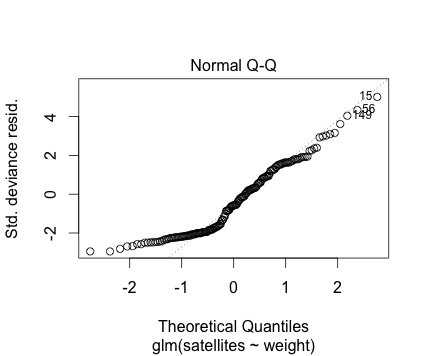

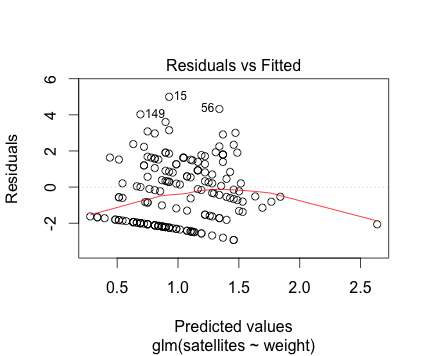
Graphical assessment of the assumptions:

ANOVA table:

| **Source of variation** | **LR Chisq** | **Df** | **Pr(>Chisq)** |
| --- | --- | --- | --- |
| **Weight** | 71.925 | 1 | < 2.20E-16 |

Coefficients:

|  | **Estimate** | **Pr(>\|t\|)** | **Coefficient** |
| --- | --- | --- | --- |
| **Intercept** | -4.28E-01 | 0.0167 | 0.651550742 |
| **Weight** | 5.89E-04 | <2e-16 | 1.000589474 |

**Summary of Appendix 8**

- Residuals: the residual vs fit plots for the GLM yield better results than those for the two transformations. The plots for both transformations show a clear fan-shape.
- Outcome of the p-value: although the value varied substantially between the general linear models and the generalised linear model, the outcome remains the same with a significant p-value for all models tested.
- Estimates of the coefficients:

Comparison of the coefficients:

|  | **Square root** | **Log(*y*+1)** | **GLM** |
| --- | --- | --- | --- |
| **Intercept** | -17.06648295 | -0.39094177 | 0.651550742 |
| **Weight** | 0.04270009 | 0.000621593 | 1.000589474 |

The table above shows that both models using transformations underestimate the values of the estimate and intercept. The square root transformation yields the largest bias to the true value of the estimate of the intercept, while the log transformation yields the largest bias to the true value of the regression coefficient (which are obtained from the generalised linear model – Stroup 2012).

**Appendix 9**

Number of car accidents based on the age of the policy holder, from McCullagh & Nelder (1983), page 298.

- “Number” refers to the number of accidents (response variable).
- “Age” refers to the age of the policy holder (explanatory variable, factor with 8 levels).

**Square root transformation**

Model:

modelCarT2<-lm((sqrt(Number))~Age, data=dataCar)


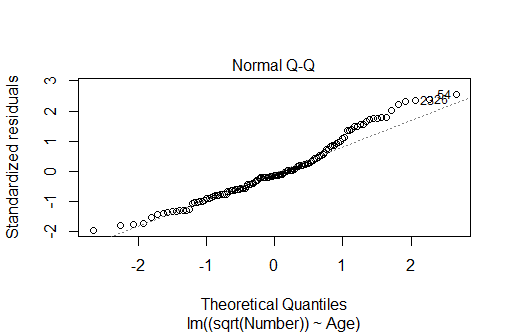

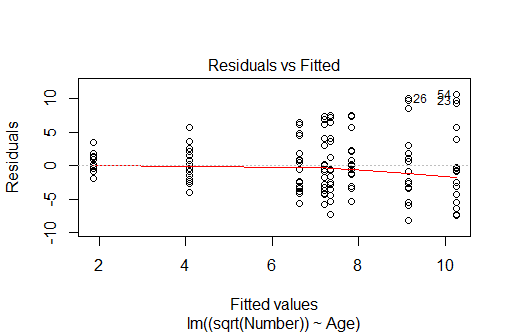
Graphical assessment of the assumptions:

ANOVA table:

| **Source of variation** | **Sum Sq** | **Df** | **F value** | **Pr(>F)** |
| --- | --- | --- | --- | --- |
| **Age** | 811.7 | 7 | 6.2711 | 2.819e-06 |
| **Residuals** | 2218.9 | 120 |  |  |

Coefficients:

|  | **Estimate** | **Pr(>\|t\|)** | **Mean** |
| --- | --- | --- | --- |
| **Age17-20 (intercept)** | 1.866 | 0.085102 | 3.481956 |
| **Age21-24** | 2.214 | 0.147959 | 16.6464 |
| **Age25-29** | 4.768 | 0.002154 | 44.00996 |
| **Age30-34** | 5.336 | 0.000633 | 51.8688 |
| **Age35-39** | 5.478 | 0.000459 | 53.93434 |
| **Age40-49** | 8.386 | 2.03e-07 | 105.1035 |
| **Age50-59** | 7.280 | 4.84e-06 | 83.64932 |
| **Age60+** | 5.974 | 0.000143 | 61.4656 |

**Logarithmic transformation**

Model:

modelCarT1<-lm((log(Number+1))~Age, data=dataCar)

Graphical assessment of the assumptions:


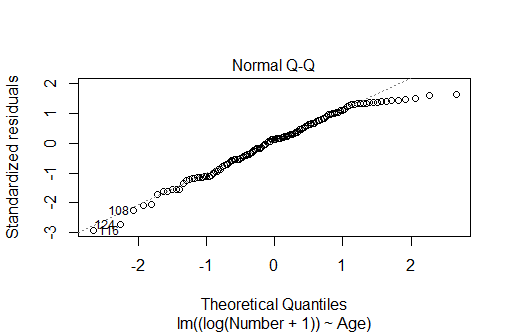

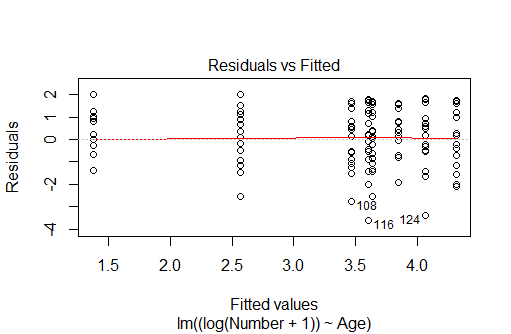


ANOVA table:

| **Source of variation** | **Sum Sq** | **Df** | **F value** | **Pr(>F)** |
| --- | --- | --- | --- | --- |
| **Age** | 102.06 | 7 | 8.9532 | 7.556e-09 |
| **Residuals** | 195.43 | 120 |  |  |

Coefficients:

|  | **Estimate** | **Pr(>\|t\|)** | **Mean** |
| --- | --- | --- | --- |
| **Age17-20 (intercept)** | 1.3730 | 3.45e-05 | 2.947174 |
| **Age21-24** | 1.1925 | 0.00932 | 12.00716 |
| **Age25-29** | 2.0953 | 8.82e-06 | 31.08216 |
| **Age30-34** | 2.2633 | 1.84e-06 | 36.95116 |
| **Age35-39** | 2.2317 | 2.49e-06 | 35.77065 |
| **Age40-49** | 2.9424 | 1.73e-09 | 73.84355 |
| **Age50-59** | 2.6939 | 2.47e-08 | 57.37572 |
| **Age60+** | 2.4759 | 0.000143 | 45.9414 |

**Generalised linear model (negative binomial distribution)**

Model:

modelCarG<-glm.nb(Number~Age. data=dataCar)


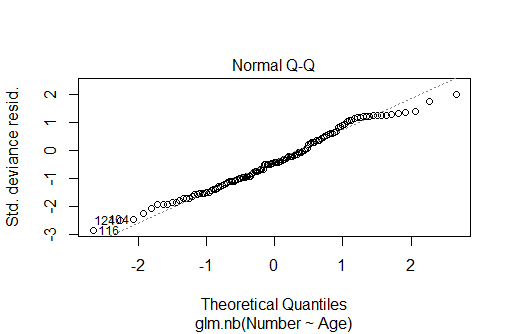

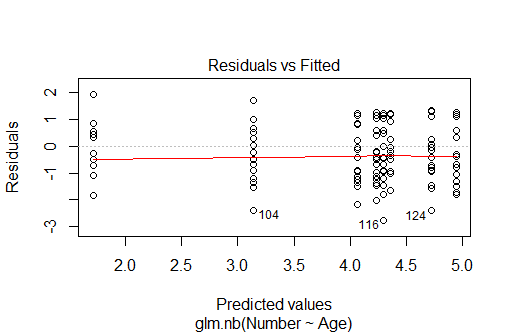
Graphical assessment of the assumptions:

ANOVA table:

| **Source of variation** | **LR Chisq** | **Df** | **Pr(>Chisq)** |
| --- | --- | --- | --- |
| **Age** | 66.641 | 7 | 7.031e-12 |

Coefficients:

|  | **Estimate** | **Pr(>\|t\|)** | **Mean** |
| --- | --- | --- | --- |
| **Age17-20 (intercept)** | 1.7160 | 3.42e-09 | 5.562235 |
| **Age21-24** | 1.4249 | 0.000369 | 23.12467 |
| **Age25-29** | 2.3465 | 3.74e-09 | 58.11943 |
| **Age30-34** | 2.5153 | 2.57e-10 | 68.80662 |
| **Age35-39** | 2.5821 | 8.50e-11 | 73.5599 |
| **Age40-49** | 3.2247 | 4.77e-16 | 139.8681 |
| **Age50-59** | 3.0019 | 4.23e-14 | 111.9329 |
| **Age60+** | 2.6391 | 3.23e-11 | 77.87461 |

**Summary of Appendix 9**

- Residuals: the residual vs fit plot for the model of the square root transformation is not optimal. However, the plots for the model using the log transformation and the generalised linear model (negative binomial) are equally good.
- Outcome of the p-value: although the value varied between the three models, the outcome remains the same with a significant p-value for each of the models tested.
- Estimates of means:

Comparison between the three models:

|  | **Square root** | **Log (*y*+1)** | **GLM** |
| --- | --- | --- | --- |
| **Age17-20 (intercept)** | 3.481956 | 2.947174 | 5.562235 |
| **Age21-24** | 16.6464 | 12.00716 | 23.12467 |
| **Age25-29** | 44.00996 | 31.08216 | 58.11943 |
| **Age30-34** | 51.8688 | 36.95116 | 68.80662 |
| **Age35-39** | 53.93434 | 35.77065 | 73.5599 |
| **Age40-49** | 105.1035 | 73.84355 | 139.8681 |
| **Age50-59** | 83.64932 | 57.37572 | 111.9329 |
| **Age60+** | 61.4656 | 45.9414 | 77.87461 |

The table above shows that both models using transformations underestimate the values of the means from each group. The log transformation yields the largest bias to the true value of the estimate (which is obtained from the generalised linear model – Stroup 2012).

**Appendix 10**

Number of car accidents based on the age of the vehicle, from McCullagh & Nelder (1983), page 298.

- “Number” refers to the number of accidents (response variable).
- “Vehicle” refers to the age of the policy holder (explanatory variable, factor with 4 levels).

**Square root transformation**

Model:

modelCarT2<-lm((sqrt(Number))~Vehicle, data=dataCar)


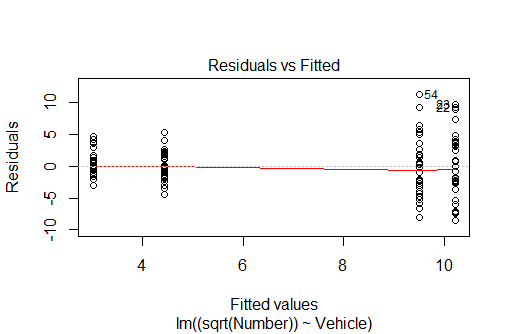

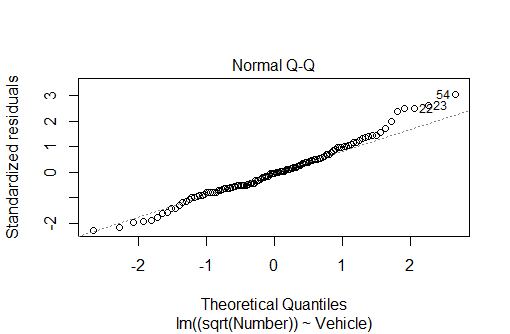
Graphical assessment of the assumptions:

ANOVA table:

| **Source of variation** | **Sum Sq** | **Df** | **F value** | **Pr(>F)** |
| --- | --- | --- | --- | --- |
| **Vehicle** | 1247.1 | 3 | 28.903 | 3.071e-14 |
| **Residuals** | 1783.5 | 124 |  |  |

Coefficients:

|  | **Estimate** | **Pr(>\|t\|)** | **Mean** |
| --- | --- | --- | --- |
| **Vehicle 0 to 3 (intercept)** | 10.2171 | < 2e-16 | 104.3891 |
| **Vehicle 4 to 7** | -0.7011 | 0.461 | 90.55426 |
| **Vehicle 8 to 9** | -5.7824 | 1.25e-08 | 19.66656 |
| **Vehicle 10+** | -7.2018 | 6.44e-12 | 9.092034 |

**Logarithmic transformation**

Model:

modelCarT1<-lm((log(Number+1))~Vehicle, data=dataCar)


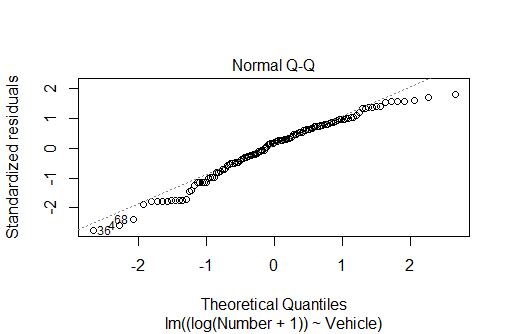

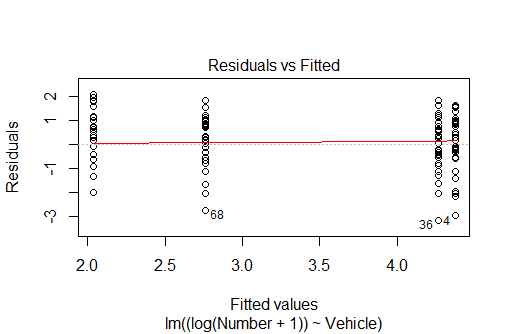
Graphical assessment of the assumptions:

ANOVA table:

| **Source of variation** | **Sum Sq** | **Df** | **F value** | **Pr(>F)** |
| --- | --- | --- | --- | --- |
| **Vehicle** | 127.01 | 3 | 30.794 | 6.015e-15 |
| **Residuals** | 170.48 | 124 |  |  |

Coefficients:

|  | **Estimate** | **Pr(>\|t\|)** | **Mean** |
| --- | --- | --- | --- |
| **Vehicle 0 to 3 (intercept)** | 4.3747 | < 2e-16 | 78.41601 |
| **Vehicle 4 to 7** | -0.1062 | 0.718 | 70.41443 |
| **Vehicle 8 to 9** | -1.6132 | 2.05e-07 | 14.82356 |
| **Vehicle 10+** | -2.3398 | 8.28e-13 | 6.651487 |

**Generalised linear model (negative binomial distribution)**

Model:

modelCarG<-glm.nb(Number~Vehicle, data=dataCar)

Graphical assessment of the assumptions:


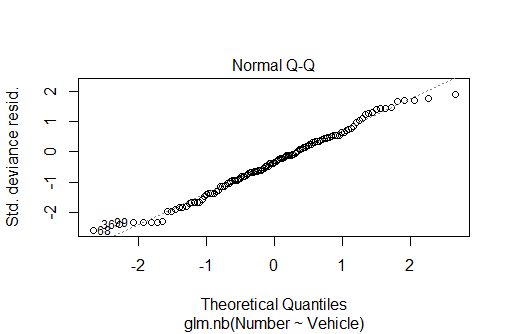

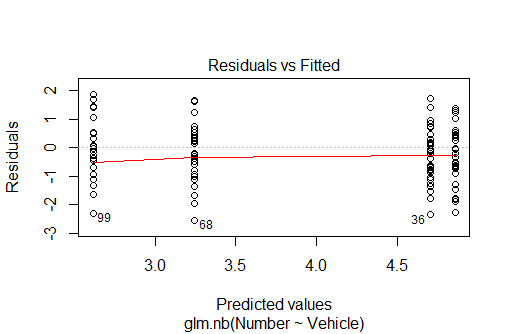


ANOVA table:

|  | **LR Chisq** | **Df** | **Pr(>Chisq)** |
| --- | --- | --- | --- |
| **Vehicle** | 95.011 | 3 | < 2.2e-16 |

Coefficients:

|  | **Estimate** | **Pr(>\|t\|)** | **Mean** |
| --- | --- | --- | --- |
| **Vehicle 0 to 3 (intercept)** | 4.8613 | < 2e-16 | 129.192 |
| **Vehicle 4 to 7** | -0.1526 | 0.548 | 110.9079 |
| **Vehicle 8 to 9** | -1.6153 | 2.79e-10 | 25.68738 |
| **Vehicle 10+** | -2.2471 | < 2e-16 | 13.65629 |

**Summary of Appendix 10**

- Residuals: the residual vs fit plot obtained for the square root transformation model is acceptable but slightly inferior to those of the two other models. For the log transformation and the generalised linear model, both models show very similar and well distributed plots.
- Outcome of the p-value: the p-value is significant in each of the three models.
- Estimates of the means:

Comparison between the three models:

|  | **Square root** | **Log(*y*+1)** | **GLM** |
| --- | --- | --- | --- |
| **Vehicle 0 to 3 (intercept)** | 104.3891 | 78.41601 | 129.192 |
| **Vehicle 4 to 7** | 90.55426 | 70.41443 | 110.9079 |
| **Vehicle 8 to 9** | 19.66656 | 14.82356 | 25.68738 |
| **Vehicle 10+** | 9.092034 | 6.651487 | 13.65629 |

The table above shows that both models using transformations underestimate the values of the means from each group. The log transformation yields the largest bias to the true value of the estimate (which is obtained from the generalised linear model – Stroup 2012).

**Appendix 11**

Number of car accidents based on the age of the vehicle and the age of the policy holder, from McCullagh & Nelder (1983), page 298.

- “Number” refers to the number of accidents (response variable).
- “Vehicle” refers to the age of the policy holder (explanatory variable, factor with 4 levels).
- “Age” refers to the age of the policy holder (explanatory variable, factor with 8 levels).

**Square root transformation**

Model:

modelCarT2<-lm((sqrt(Number))~Vehicle*Age, data=dataCar)


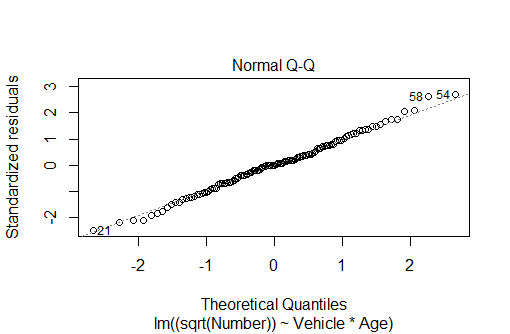

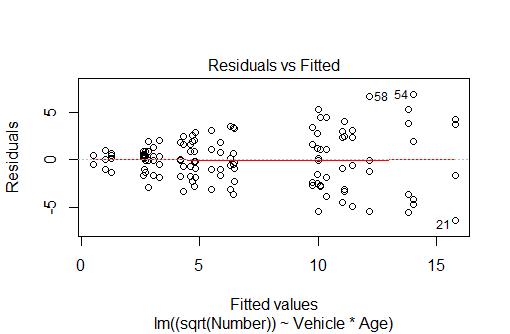
Graphical assessment of the assumptions:

ANOVA table (type II):

| **Source of variation** | **Sum Sq** | **Df** | **F value** | **Pr(>F)** |
| --- | --- | --- | --- | --- |
| **Vehicle** | 1247.10 | 3 | 48.4931 | < 2.2e-16 |
| **Age** | 811.70 | 7 | 13.5268 | 4.746e-12 |
| **Vehicle:Age** | 148.82 | 21 | 0.8267 | 0.6813 |
| **Residuals** | 822.95 | 96 |  |  |

Coefficients:

|  | **Estimate** | **Pr(>\|t\|)** | **Mean** |
| --- | --- | --- | --- |
| **Intercept (Vehicle 0 to 3 and Age 17-20)** | 2.680689 | 0.070179 | 7.186094 |
| **Vehicle10+** | -2.18069 | 0.294841 | 0.249999 |
| **Vehicle4 to 7** | 0.604235 | 0.771026 | 10.79073 |
| **Vehicle8 to 9** | -1.68069 | 0.418912 | 0.999998 |
| **Age21-24** | 3.183315 | 0.127436 | 34.38654 |
| **Age25-29** | 7.298331 | 0.000650 | 99.58084 |
| **Age30-34** | 8.36187 | 0.000108 | 121.9381 |
| **Age35-39** | 8.763762 | 5.28e-05 | 130.9755 |
| **Age40-49** | 13.09735 | 7.96e-09 | 248.9465 |
| **Age50-59** | 11.14219 | 5.21e-07 | 191.072 |
| **Age60+** | 8.444668 | 9.34e-05 | 123.7736 |
| **Vehicle10+:Age21-24** | -2.39675 | 0.415042 | 0.080621 |
| **Vehicle4 to 7:Age21-24** | -0.0049 | 0.998669 | 7.159847 |
| **Vehicle8 to 9:Age21-24** | -1.47637 | 0.615243 | 1.450384 |
| **Vehicle10+:Age25-29** | -5.18463 | 0.079770 | 6.269721 |
| **Vehicle4 to 7:Age25-29** | -0.84327 | 0.773957 | 3.376109 |
| **Vehicle8 to 9:Age25-29** | -4.09452 | 0.165195 | 1.998918 |
| **Vehicle10+:Age30-34** | -5.80257 | 0.050356 | 9.746141 |
| **Vehicle4 to 7:Age30-34** | -1.54617 | 0.598656 | 1.287133 |
| **Vehicle8 to 9:Age30-34** | -4.75596 | 0.107574 | 4.30675 |
| **Vehicle10+:Age35-39** | -6.42228 | 0.030685 | 13.9995 |
| **Vehicle4 to 7:Age35-39** | -1.71228 | 0.560039 | 0.937816 |
| **Vehicle8 to 9:Age35-39** | -5.00832 | 0.090390 | 5.417866 |
| **Vehicle10+:Age40-49** | -8.78621 | 0.003430 | 37.27739 |
| **Vehicle4 to 7:Age40-49** | -2.37753 | 0.418780 | 0.091905 |
| **Vehicle8 to 9:Age40-49** | -7.68124 | 0.010124 | 25.00551 |
| **Vehicle10+:Age50-59** | -7.33473 | 0.013924 | 21.6601 |
| **Vehicle4 to 7:Age50-59** | -2.25446 | 0.443189 | 0.181671 |
| **Vehicle8 to 9:Age50-59** | -5.8586 | 0.048218 | 10.09912 |
| **Vehicle10+:Age60+** | -4.24158 | 0.150681 | 2.436381 |
| **Vehicle4 to 7:Age60+** | -1.70368 | 0.562008 | 0.954547 |
| **Vehicle8 to 9:Age60+** | -3.9389 | 0.181693 | 1.583095 |

**Logarithmic transformation**

Model:

modelCarT1<-lm((log(Number+1))~Vehicle*Age, data=dataCar)


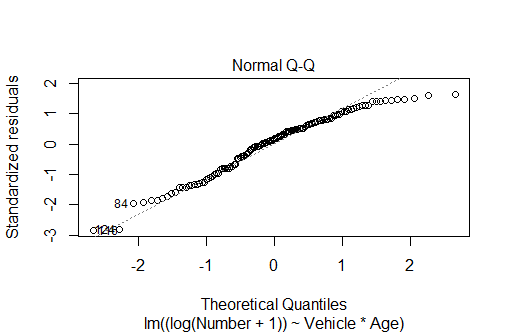

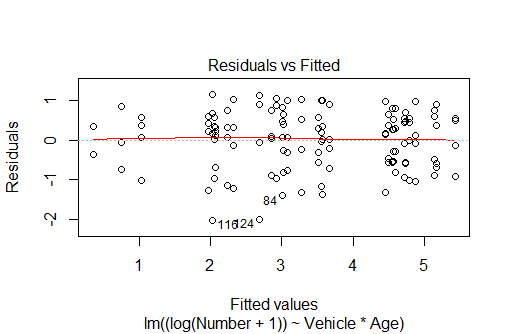
Graphical assessment of the assumptions:

ANOVA table (type II):

| **Source of variation** | **Sum Sq** | **Df** | **F value** | **Pr(>F)** |
| --- | --- | --- | --- | --- |
| **Vehicle** | 127.012 | 3 | 62.2644 | <2e-16 |
| **Age** | 102.065 | 7 | 21.4435 | <2e-16 |
| **Vehicle:Age** | 3.138 | 21 | 0.2198 | 0.9999 |
| **Residuals** | 65.276 | 96 |  |  |

Coefficients:

|  | **Estimate** | **Pr(>\|t\|)** | **Mean** |
| --- | --- | --- | --- |
| **Intercept (Vehicle 0 to 3 and Age 17-20)** | 2.07100 | 2.35e-06 | 6.932752 |
| **Vehicle10+** | -1.72443 | 0.0039 | 0.414208 |
| **Vehicle4 to 7** | 0.25455 | 0.6634 | 9.232306 |
| **Vehicle8 to 9** | -1.32207 | 0.0256 | 1.114736 |
| **Age21-24** | 1.44508 | 0.0149 | 32.65225 |
| **Age25-29** | 2.49641 | 4.40e-05 | 95.29438 |
| **Age30-34** | 2.65884 | 1.51e-05 | 112.2774 |
| **Age35-39** | 2.72426 | 9.70e-06 | 119.9358 |
| **Age40-49** | 3.36611 | 9.55e-08 | 228.7772 |
| **Age50-59** | 3.06827 | 8.66e-07 | 169.5912 |
| **Age60+** | 2.67055 | 1.40e-05 | 113.6117 |
| **Vehicle10+:Age21-24** | -0.76807 | 0.3540 | 2.680063 |
| **Vehicle4 to 7:Age21-24** | -0.10219 | 0.9016 | 6.162148 |
| **Vehicle8 to 9:Age21-24** | -0.14026 | 0.8653 | 5.89461 |
| **Vehicle10+:Age25-29** | -0.87968 | 0.2887 | 2.291423 |
| **Vehicle4 to 7:Age25-29** | -0.32746 | 0.6922 | 4.717548 |
| **Vehicle8 to 9:Age25-29** | -0.39744 | 0.6309 | 4.331113 |
| **Vehicle10+:Age30-34** | -0.76495 | 0.3559 | 2.691563 |
| **Vehicle4 to 7:Age30-34** | -0.43259 | 0.6011 | 4.146979 |
| **Vehicle8 to 9:Age30-34** | -0.38461 | 0.6420 | 4.399952 |
| **Vehicle10+:Age35-39** | -1.05000 | 0.2060 | 1.775969 |
| **Vehicle4 to 7:Age35-39** | -0.45864 | 0.5794 | 4.014632 |
| **Vehicle8 to 9:Age35-39** | -0.46141 | 0.5771 | 4.00076 |
| **Vehicle10+:Age40-49** | -0.63643 | 0.4421 | 3.19784 |
| **Vehicle4 to 7:Age40-49** | -0.51755 | 0.5317 | 3.727753 |
| **Vehicle8 to 9:Age40-49** | -0.54066 | 0.5136 | 3.619747 |
| **Vehicle10+:Age50-59** | -0.72875 | 0.3790 | 2.827646 |
| **Vehicle4 to 7:Age50-59** | -0.51402 | 0.5345 | 3.744471 |
| **Vehicle8 to 9:Age50-59** | -0.25475 | 0.7580 | 5.148757 |
| **Vehicle10+:Age60+** | -0.09525 | 0.9083 | 6.212027 |
| **Vehicle4 to 7:Age60+** | -0.53351 | 0.5192 | 3.652897 |
| **Vehicle8 to 9:Age60+** | -0.14981 | 0.8562 | 5.82908 |

**Generalised linear model (negative binomial distribution)**

Model:

modelCarG<-glm.nb(Number~Vehicle*Age, data=dataCar)


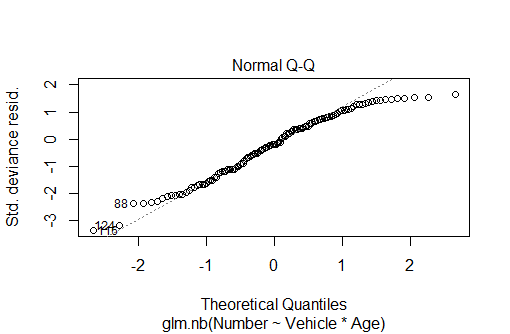

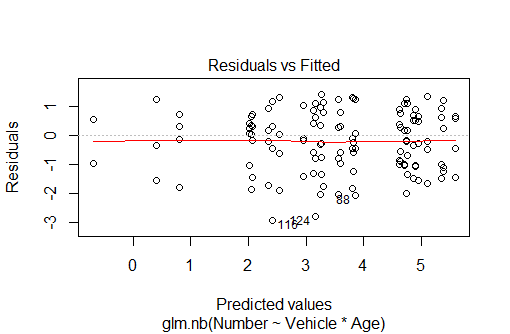
Graphical assessment of the assumptions:

ANOVA table (type II):

|  | **LR Chisq** | **Df** | **Pr(>Chisq)** |
| --- | --- | --- | --- |
| **Vehicle** | 236.639 | 3 | <2e-16 |
| **Age** | 186.312 | 7 | <2e-16 |
| **Vehicle:Age** | 9.768 | 21 | 0.9818 |

Coefficients:

|  | **Estimate** | **Pr(>\|t\|)** | **Mean** |
| --- | --- | --- | --- |
| **Intercept (Vehicle 0 to 3 and Age 17-20)** | 2.01490 | 3.81e-08 | 7.499977 |
| **Vehicle10+** | -2.70805 | 0.00159 | 0.499999 |
| **Vehicle4 to 7** | 0.53063 | 0.29310 | 12.74998 |
| **Vehicle8 to 9** | -1.60944 | 0.01111 | 1.499992 |
| **Age21-24** | 1.57554 | 0.00136 | 36.25002 |
| **Age25-29** | 2.62949 | 6.84e-08 | 103.9999 |
| **Age30-34** | 2.85838 | 4.33e-09 | 130.7491 |
| **Age35-39** | 2.93208 | 1.70e-09 | 140.7493 |
| **Age40-49** | 3.57422 | 1.89e-13 | 267.5001 |
| **Age50-59** | 3.34404 | 6.02e-12 | 212.4996 |
| **Age60+** | 2.88480 | 3.11e-09 | 134.2495 |
| **Vehicle10+:Age21-24** | -0.07146 | 0.94453 | 6.98273 |
| **Vehicle4 to 7:Age21-24** | -0.29243 | 0.66933 | 5.598339 |
| **Vehicle8 to 9:Age21-24** | 0.09844 | 0.90220 | 8.275836 |
| **Vehicle10+:Age25-29** | 0.11135 | 0.91005 | 8.38337 |
| **Vehicle4 to 7:Age25-29** | -0.55496 | 0.41392 | 4.305701 |
| **Vehicle8 to 9:Age25-29** | -0.07744 | 0.92159 | 6.941098 |
| **Vehicle10+:Age30-34** | 0.18614 | 0.84953 | 9.034404 |
| **Vehicle4 to 7:Age30-34** | -0.69439 | 0.30628 | 3.745331 |
| **Vehicle8 to 9:Age30-34** | -0.12835 | 0.87013 | 6.596571 |
| **Vehicle10+:Age35-39** | 0.18143 | 0.85316 | 8.991952 |
| **Vehicle4 to 7:Age35-39** | -0.71972 | 0.28884 | 3.651653 |
| **Vehicle8 to 9:Age35-39** | -0.08911 | 0.90953 | 6.860566 |
| **Vehicle10+:Age40-49** | 0.38660 | 0.69123 | 11.03972 |
| **Vehicle4 to 7:Age40-49** | -0.73411 | 0.27835 | 3.599482 |
| **Vehicle8 to 9:Age40-49** | -0.11895 | 0.87893 | 6.658871 |
| **Vehicle10+:Age50-59** | 0.50611 | 0.60334 | 12.44116 |
| **Vehicle4 to 7:Age50-59** | -0.77307 | 0.25394 | 3.461943 |
| **Vehicle8 to 9:Age50-59** | 0.05716 | 0.94167 | 7.941165 |
| **Vehicle10+:Age60+** | 1.11340 | 0.25281 | 22.83513 |
| **Vehicle4 to 7:Age60+** | -0.68976 | 0.30946 | 3.762712 |
| **Vehicle8 to 9:Age60+** | 0.27927 | 0.72121 | 9.916202 |

**Summary of Appendix 11**

- Residuals: the residual vs fit plot obtained for the square root transformation model is not acceptable as it shows a clear cone shape. Normally, the analysis would be stopped at this point given that the assumptions are not met with this model. In the present case we carried on with the analysis to contrast the results. Yet this is not recommended. For the log transformation and the generalised linear model, both show very similar and acceptable plots.
- Outcome of the p-value: the decision based on the p-value does not change among all three models tested.
- Estimates of the means:

Comparison between the three models:

|  | **Square root** | **Log(*y*+1)** | **GLM** |
| --- | --- | --- | --- |
| **Intercept (Vehicle 0 to 3 and Age 17-20)** | 7.186094 | 6.932752 | 7.499977 |
| **Vehicle10+** | 0.249999 | 0.414208 | 0.499999 |
| **Vehicle4 to 7** | 10.79073 | 9.232306 | 12.74998 |
| **Vehicle8 to 9** | 0.999998 | 1.114736 | 1.499992 |
| **Age21-24** | 34.38654 | 32.65225 | 36.25002 |
| **Age25-29** | 99.58084 | 95.29438 | 103.9999 |
| **Age30-34** | 121.9381 | 112.2774 | 130.7491 |
| **Age35-39** | 130.9755 | 119.9358 | 140.7493 |
| **Age40-49** | 248.9465 | 228.7772 | 267.5001 |
| **Age50-59** | 191.072 | 169.5912 | 212.4996 |
| **Age60+** | 123.7736 | 113.6117 | 134.2495 |
| **Vehicle10+:Age21-24** | 0.080621 | 2.680063 | 6.98273 |
| **Vehicle4 to 7:Age21-24** | 7.159847 | 6.162148 | 5.598339 |
| **Vehicle8 to 9:Age21-24** | 1.450384 | 5.89461 | 8.275836 |
| **Vehicle10+:Age25-29** | 6.269721 | 2.291423 | 8.38337 |
| **Vehicle4 to 7:Age25-29** | 3.376109 | 4.717548 | 4.305701 |
| **Vehicle8 to 9:Age25-29** | 1.998918 | 4.331113 | 6.941098 |
| **Vehicle10+:Age30-34** | 9.746141 | 2.691563 | 9.034404 |
| **Vehicle4 to 7:Age30-34** | 1.287133 | 4.146979 | 3.745331 |
| **Vehicle8 to 9:Age30-34** | 4.30675 | 4.399952 | 6.596571 |
| **Vehicle10+:Age35-39** | 13.9995 | 1.775969 | 8.991952 |
| **Vehicle4 to 7:Age35-39** | 0.937816 | 4.014632 | 3.651653 |
| **Vehicle8 to 9:Age35-39** | 5.417866 | 4.00076 | 6.860566 |
| **Vehicle10+:Age40-49** | 37.27739 | 3.19784 | 11.03972 |
| **Vehicle4 to 7:Age40-49** | 0.091905 | 3.727753 | 3.599482 |
| **Vehicle8 to 9:Age40-49** | 25.00551 | 3.619747 | 6.658871 |
| **Vehicle10+:Age50-59** | 21.6601 | 2.827646 | 12.44116 |
| **Vehicle4 to 7:Age50-59** | 0.181671 | 3.744471 | 3.461943 |
| **Vehicle8 to 9:Age50-59** | 10.09912 | 5.148757 | 7.941165 |
| **Vehicle10+:Age60+** | 2.436381 | 6.212027 | 22.83513 |
| **Vehicle4 to 7:Age60+** | 0.954547 | 3.652897 | 3.762712 |
| **Vehicle8 to 9:Age60+** | 1.583095 | 5.82908 | 9.916202 |

The pattern is not very clear in this table. Although one would normally focus only on the estimates from the individual factors (since the interaction term is not significant), we can observe that the estimates for the combinations of factors are not following a clear trend. Some of the values from the models using transformations are underestimated, while other are overestimated compared to the values from the generalised linear model.

**Appendix 12**

Number of satellite horseshoe crab males relative to female crab colour, from Agresti (1996), page 76, where:

- “Satellites” refers to the number of satellites ( response variable)
- “Colour” refers to the colour of female crabs ( explanatory variable, factor with 4 levels)

**Square root transformation**

Model:


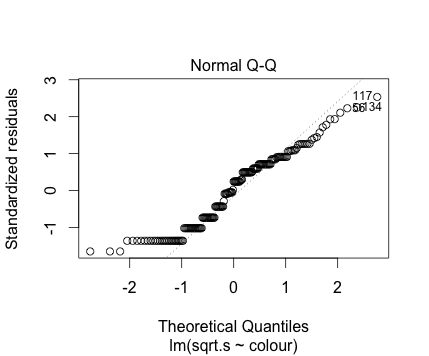

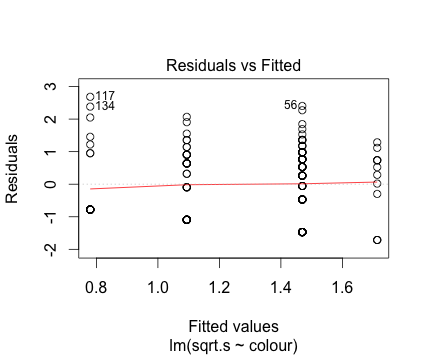
model1<-lm(sqrt.satellites~colour, data=crabdata)

Graphical assessment of the assumptions:

ANOVA table:

| **Source of variation** | **Sum Sq** | **Df** | **F value** | **Pr(>F)** |
| --- | --- | --- | --- | --- |
| **Colour** | 12.603 | 3 | 3.5752 | 0.01525 |
| **Residuals** | 198.577 | 169 |  |  |

Coefficients:

|  | **Estimate** | **Pr(>\|t\|)** | **Mean** |
| --- | --- | --- | --- |
| **Colour 2 (Intercept)** | 1.7133 | 1.56e-7 | 2.93539689 |
| **Colour 3** | -0.2437 | 0.4641 | 2.15972416 |
| **Colour 4** | -0.2437 | 0.0812 | 2.15972416 |
| **Colour 5** | -0.9335 | 0.0175 | 0.60808804 |

**Logarithmic transformation**

Model:

model2<-lm(log(satellites+1)~colour, data=crabdata)


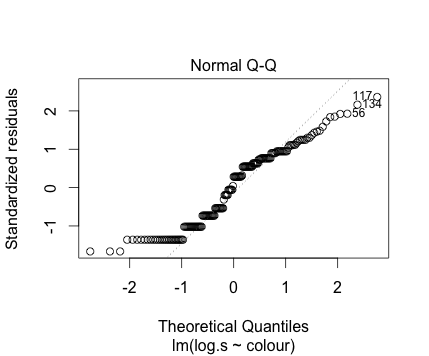

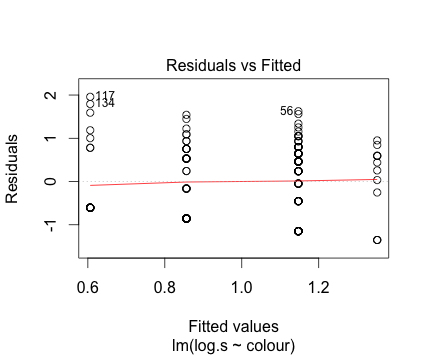
Graphical assessment of the assumptions:

ANOVA table:

| **Source of variation** | **Sum Sq** | **Df** | **F value** | **Pr(>F)** |
| --- | --- | --- | --- | --- |
| **Colour** | 7.808 | 3 | 3.6177 | 0.01443 |
| **Residuals** | 121.581 | 169 |  |  |

Coefficients:

|  | **Estimate** | **Pr(>\|t\|)** | **Mean** |
| --- | --- | --- | --- |
| **Colour 2 (Intercept)** | 1.3520 | 1.25e-7 | 2.865148102 |
| **Colour 3** | -0.2049 | 0.4316 | 2.149047418 |
| **Colour 4** | -0.4958 | 0.0744 | 1.354197723 |
| **Colour 5** | -0.7459 | 0.0153 | 0.833267695 |

**Generalised linear model (Poisson distribution)**

Model:


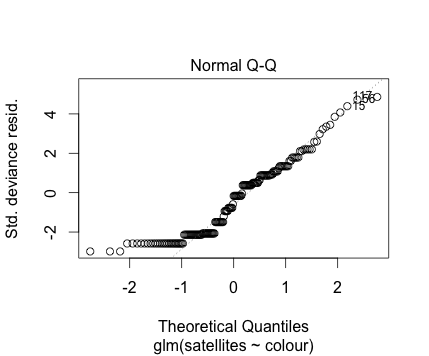

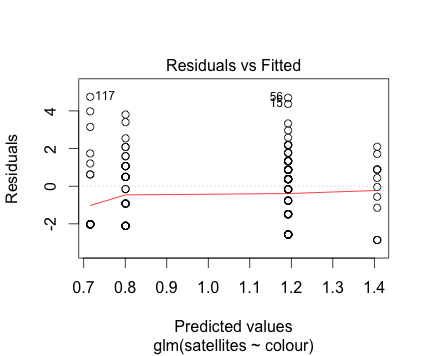
model3<-glm(satellites~colour, family=poisson, data=crabdata)

Graphical assessment of the assumptions:

ANOVA table:

| **Source of variation** | **LR Chisq** | **Df** | **Pr(>Chisq)** |
| --- | --- | --- | --- |
| **Colour** | 23.653 | 3 | 2.952e-5 |

Coefficients:

|  | **Estimate** | **Pr(>\|t\|)** | **Mean** |
| --- | --- | --- | --- |
| **Colour 2 (Intercept)** | 1.4069 | 2e-16 | 4.083277603 |
| **Colour 3** | -0.2146 | 0.162487 | 3.294650195 |
| **Colour 4** | -0.6061 | 0.000532 | 2.227322074 |
| **Colour 5** | -0.6913 | 0.000814 | 2.045413562 |

**Summary of Appendix 12**

- Residuals: the residual vs fit plots for all three models are acceptable.
- Outcome of the p-value: although the value varied substantially between the linear models using transformed data and the generalised linear model, the outcome remains the same with a significant p-value for each of the models tested.
- Estimates of coefficients:

Comparison of the means:

|  | **Square root** | **Log(*y*+1)** | **GLM** |
| --- | --- | --- | --- |
| **Colour 2 (intercept)** | 2.93539689 | 2.865148102 | 4.083277603 |
| **Colour 3** | 2.15972416 | 2.149047418 | 3.294650195 |
| **Colour 4** | 2.15972416 | 1.354197723 | 2.227322074 |
| **Colour 5** | 0.60808804 | 0.833267695 | 2.045413562 |

The table above shows that both models using transformations underestimate the values of the means from each group. The log transformation yields the largest bias to the true value of the estimate (which is obtained from the generalised linear model – Stroup 2012).

**Appendix 13**

Number of satellite horseshoe crab males relative to female spine condition, from Agresti (1996), page 76, where:

- “Satellites” refers to the number of satellites ( response variable)
- “Spine” refers to the spine condition of female crabs (explanatory variable factor with 3 levels)

**Square root transformation**

Model:


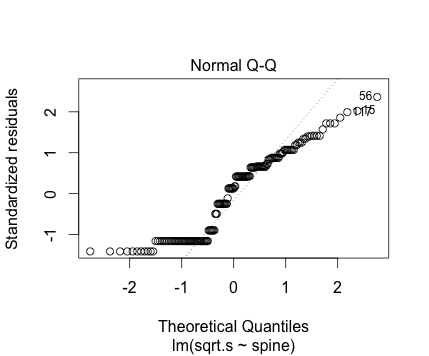

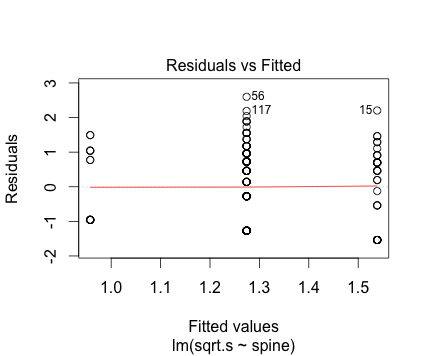
model1<-lm(sqrt.satellites~spine, data=crabdata)

Graphical assessment of the assumptions:

ANOVA table:

| **Source of variation** | **Sum Sq** | **Df** | **F value** | **Pr(>F)** |
| --- | --- | --- | --- | --- |
| **Spine** | 3.943 | 2 | 1.6171 | 0.2015 |
| **Residuals** | 207.237 | 170 |  |  |

Coefficients:

|  | **Estimate** | **Pr(>\|t\|)** | **Mean** |
| --- | --- | --- | --- |
| **Spine 1 (Intercept)** | 1.5385 | 1.08E-14 | 2.36698225 |
| **Spine 2** | -0.5809 | 0.0874 | 0.91699776 |
| **Spine 3** | -0.2643 | 0.2043 | 1.62358564 |

**Logarithmic transformation**

Model:


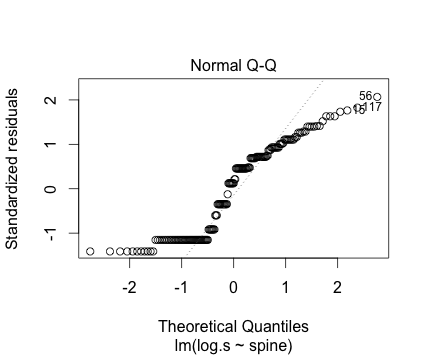

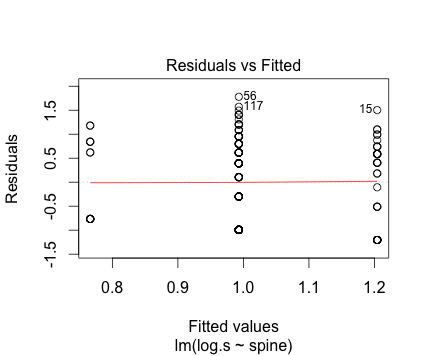
model2<-lm(log(satellites+1)~spine, data=crabdata)

Graphical assessment of the assumptions:

ANOVA table:

| **Source of variation** | **Sum Sq** | **Df** | **F value** | **Pr(>F)** |
| --- | --- | --- | --- | --- |
| **Spine** | 2.31 | 2 | 1.5448 | 0.2163 |
| **Residuals** | 127.08 | 170 |  |  |

Coefficients:

|  | **Estimate** | **Pr(>\|t\|)** | **Mean** |
| --- | --- | --- | --- |
| **Spine 1 (Intercept)** | 1.2042 | 1.10E-14 | 3.657598631 |
| **Spine 2** | -0.438 | 0.0997 | 1.605435918 |
| **Spine 3** | -0.2112 | 0.1953 | 2.575839594 |

**Generalised linear model (Poisson distribution)**

Model:

model3<-glm(satellites~spine, family=poisson, data=crabdata)


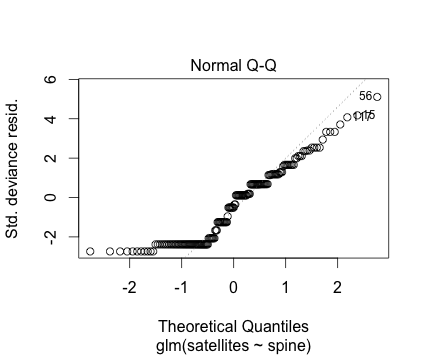

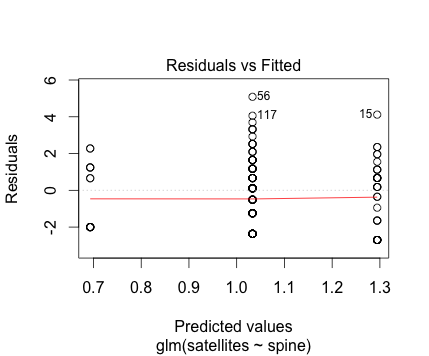
Graphical assessment of the assumptions:

ANOVA table:

| **Source of variation** | **LR Chisq** | **Df** | **Pr(>Chisq)** |
| --- | --- | --- | --- |
| **Spine** | 11.631 | 2 | 0.002981 |

Coefficients:

|  | **Estimate** | **Pr(>\|t\|)** | **Mean** |
| --- | --- | --- | --- |
| **Spine 1 (Intercept)** | 1.29436 | < 2e-16 | 3.648660084 |
| **Spine 2** | -0.60121 | 0.00289 | 2.000005639 |
| **Spine 3** | -0.2612 | 0.01024 | 2.809931203 |

**Summary of Appendix 13**

- Residuals: the residual vs fit plots for all three models are acceptable.
- Outcome of the p-value: the p-values for the two linear models applied to transformed data are non-significant, while the p-value of the generalised linear model is significant.
- Estimates of the coefficients:

Comparison of the means:

|  | **Square root** | **Log(*y*+1)** | **GLM** |
| --- | --- | --- | --- |
| **Spine 1 (intercept)** | 2.36698225 | 3.657598631 | 3.648660084 |
| **Spine 2** | 0.91699776 | 1.605435918 | 2.000005639 |
| **Spine 3** | 1.62358564 | 2.575839594 | 2.809931203 |

The table above shows that both models using transformations underestimate the values of the means from each group. The square root transformation yields the largest bias to the true value of the estimate (which is obtained from the generalised linear model – Stroup 2012).

**Appendix 14**

Number of satellite horseshoe crab males relative to female colour and spine condition, from Agresti (1996), page 76, where:

- “Satellites” refers to the number of satellites ( response variable)
- “Colour” refers to the colour of female crabs (explanatory variable, factor with 4 levels)
- “Spine” refers to the spine condition of female crabs (explanatory variable, factor with 3 levels)

**Square root transformation**

Model:

model1<-lm(sqrt.satellites~colour*spine, data=crabdata)


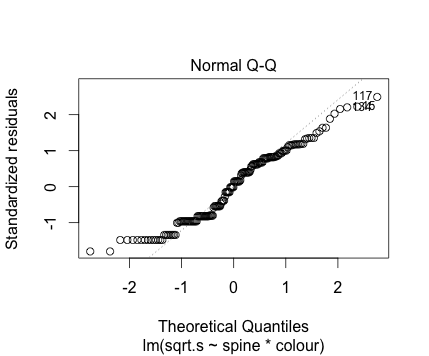

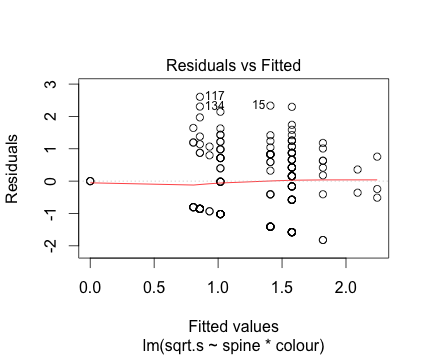
Graphical assessment of the assumptions:

ANOVA table:

| **Source of variation** | **Sum Sq** | **Df** | **F value** | **Pr(>F)** |
| --- | --- | --- | --- | --- |
| **Spine** | 2.639 | 2 | 1.1464 | 0.32035 |
| **Colour** | 11.299 | 3 | 3.2725 | 0.02271 |
| **Spine:Colour** | 10.641 | 6 | 1.541 | 0.16799 |
| **Residuals** | 185.297 | 161 |  |  |

Coefficients:

|  | **Estimate** | **Pr(>\|t\|)** | **Mean** |
| --- | --- | --- | --- |
| **(Intercept)** | 1.8197 | 9.95E-07 | 3.31130809 |
| **Spine 2** | 0.271 | 0.747 | 4.37102649 |
| **Spine 3** | -1.8197 | 0.1095 | 0 |
| **Colour 3** | -0.4108 | 0.3287 | 1.98499921 |
| **Colour 4** | 0.4243 | 0.5539 | 5.035536 |
| **Colour 5** | -1.8197 | 0.1095 | 0 |
| **Spine 2:Colour 3** | -0.8737 | 0.3571 | 0.894916 |
| **Spine 3:Colour 3** | 1.9878 | 0.0885 | 14.49705625 |
| **Spine 2:Colour 4** | -1.582 | 0.1791 | 0.05650129 |
| **Spine 3:Colour 4** | 0.5938 | 0.6488 | 5.82498225 |
| **Spine 2:Colour 5** | -0.271 | 0.876 | 2.39847169 |
| **Spine 3:Colour 5** | 2.6775 | 0.0915 | 20.22480784 |

**Logarithmic transformation**

Model:

model2<-lm(log(satellites+1)~colour*spine, data=crabdata)


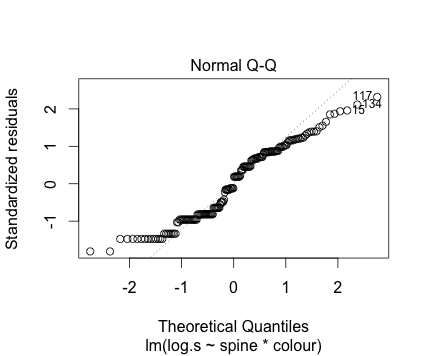

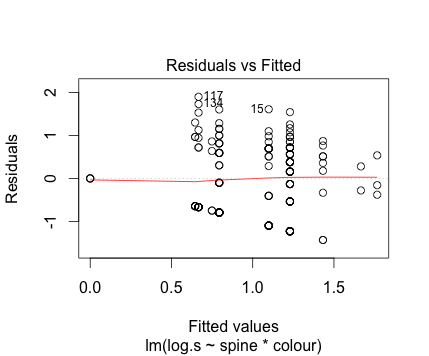
Graphical assessment of the assumptions:

ANOVA table:

| **Source of variation** | **Sum Sq** | **Df** | **F value** | **Pr(>F)** |
| --- | --- | --- | --- | --- |
| **Spine** | 1.446 | 2 | 1.0251 | 0.36109 |
| **Colour** | 6.944 | 3 | 3.2827 | 0.02241 |
| **Spine:Colour** | 6.613 | 6 | 1.5632 | 0.16113 |
| **Residuals** | 113.522 | 161 |  |  |

Coefficients:

|  | **Estimate** | **Pr(>\|t\|)** | **Mean** |
| --- | --- | --- | --- |
| **(Intercept)** | 1.4324 | 8.73E-07 | 3.188740114 |
| **Spine 2** | 0.2337 | 0.722 | 4.291490689 |
| **Spine 3** | -1.4324 | 0.108 | 0 |
| **Colour 3** | -0.3338 | 0.311 | 1.999963134 |
| **Colour 4** | 0.3337 | 0.552 | 4.848001623 |
| **Colour 5** | -1.4324 | 0.108 | 0 |
| **Spine 2:Colour 3** | -0.6867 | 0.355 | 1.10791646 |
| **Spine 3:Colour 3** | 1.5631 | 0.087 | 18.99535507 |
| **Spine 2:Colour 4** | -1.2509 | 0.175 | 0.199014537 |
| **Spine 3:Colour 4** | 0.4602 | 0.652 | 5.636601432 |
| **Spine 2:Colour 5** | -0.2337 | 0.863 | 2.315803575 |
| **Spine 3:Colour 5** | 2.0991 | 0.091 | 33.17519197 |

**Generalised linear model (Poisson distribution)**

Model:

model3<-glm(satellites~colour*spine, family=poisson, data=crabdata)


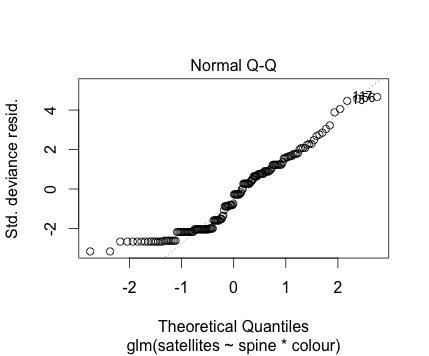

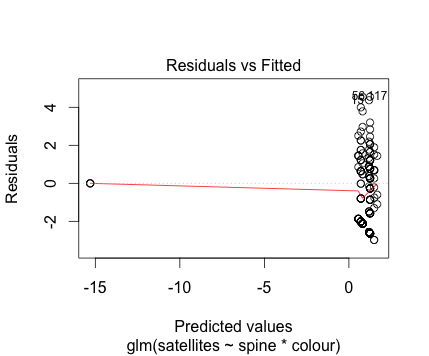
Graphical assessment of the assumptions:

ANOVA table:

| **Source of variation** | **LR Chisq** | **Df** | **Pr(>Chisq)** |
| --- | --- | --- | --- |
| **Spine** | 6.946 | 2 | 0.0310241 |
| **Colour** | 18.967 | 3 | 0.0002777 |
| **Spine:Colour** | 28.325 | 6 | 8.16E-05 |

Coefficients:

|  | **Estimate** | **Pr(>\|t\|)** | **Mean** |
| --- | --- | --- | --- |
| **(Intercept)** | 1.49165 | <2e-16 | 4.44442277 |
| **Spine 2** | 0.01242 | 0.9731 | 4.499966715 |
| **Spine 3** | -16.79424 | 0.9895 | 2.26032E-07 |
| **Colour 3** | -0.30026 | 0.1218 | 3.291653427 |
| **Colour 4** | 0.18232 | 0.5377 | 5.333299021 |
| **Colour 5** | -16.79424 | 0.9895 | 2.26032E-07 |
| **Spine 2:Colour 3** | -0.6442 | 0.1698 | 2.333688353 |
| **Spine 3:Colour 3** | 16.85334 | 0.9895 | 92710287.44 |
| **Spine 2:Colour 4** | -1.12678 | 0.0538 | 1.440326753 |
| **Spine 3:Colour 4** | 15.82683 | 0.9901 | 33213932.52 |
| **Spine 2:Colour 5** | -0.01242 | 1 | 4.389564415 |
| **Spine 3:Colour 5** | 32.90776 | 0.9854 | 8.69909E+14 |

**Summary of Appendix 14**

- Residuals: the residual vs fit plots for all three models are acceptable.
- Outcome of the p-value: although the value varied substantially between the general linear models and the generalised linear model, the outcome stayed the same with a significant p-value for each of the models tested.
- Estimates of the coefficients:

Comparison of the means:

|  | **Square root** | **Log(*y*+1)** | **GLM** |
| --- | --- | --- | --- |
| **(Intercept)** | 3.31130809 | 3.188740114 | 4.44442277 |
| **Spine 2** | 4.37102649 | 4.291490689 | 4.499966715 |
| **Spine 3** | 0 | 0 | 2.26032E-07 |
| **Colour 3** | 1.98499921 | 1.999963134 | 3.291653427 |
| **Colour 4** | 5.035536 | 4.848001623 | 5.333299021 |
| **Colour 5** | 0 | 0 | 2.26032E-07 |
| **Spine 2:Colour 3** | 0.894916 | 1.10791646 | 2.333688353 |
| **Spine 3:Colour 3** | 14.49705625 | 18.99535507 | 92710287.44 |
| **Spine 2:Colour 4** | 0.05650129 | 0.199014537 | 1.440326753 |
| **Spine 3:Colour 4** | 5.82498225 | 5.636601432 | 33213932.52 |
| **Spine 2:Colour 5** | 2.39847169 | 2.315803575 | 4.389564415 |
| **Spine 3:Colour 5** | 20.22480784 | 33.17519197 | 8.69909E+14 |

The table above shows that both models using transformations underestimate the values of the means from each group. The square root transformation most often yields the largest bias to the true value of the estimate (which is obtained from the generalised linear model – Stroup 2012), although this varies between factors and their interactions.

**Appendix 15**

**Template of R script** used in the comparisons between transformations (square root and log(y+1)) and generalised linear models.

Linear model on square root transformed data:

model_sqrt<-lm((sqrt(Response))~Variable, data=dataset)

fits <- fitted(model_sqrt)

res <- residuals(model_sqrt)

plot(model_sqrt) # to obtain the residual vs. fit plot, and the normal QQ plot

library("car")

Anova(model_sqrt, type="II")

summary(model_sqrt)

Linear model on log transformed data:

model_log<-lm((log(Response+1))~Variable, data=dataset)

fits <- fitted(model_log)

res <- residuals(model_log)

plot(model_log) # to obtain the residual vs. fit plot, and the normal QQ plot

library("car")

Anova(model_log, type="II")

summary(model_log)

Generalised linear model:

model_GLM<-glm(Response~Variable, family=poisson, data=dataset)

fits <- fitted(model_GLM)

res <- residuals(model_GLM)

plot(model_GLM)

library("car")

Anova(model_GLM, type="II")

summary(model_GLM)
